# ToF-SIMS evaluation of PEG-related mass peaks and applications in PEG detection in cosmetic products

**DOI:** 10.1038/s41598-024-65504-4

**Published:** 2024-07-01

**Authors:** Yanjie Shen, Jiyoung Son, Xiao-Ying Yu

**Affiliations:** 1 College of Biology and Oceanography, Weifang University, 5147 Dongfeng East Street, Weifang, 261061 Shandong China; 2 Materials Science and Technology Division, Oak Ridge National Laboratory, Oak Ridge, TN 37830 USA; 3 Energy and Environment Directorate, Pacific Northwest National Laboratory, Richland, WA 99354 USA

**Keywords:** Polyethylene glycols (PEGs), Environmental polymer, Spectral signature, Principal component analysis (PCA), Cosmetic product, ToF-SIMS, Atmospheric chemistry, Environmental monitoring, Pollution remediation, Mass spectrometry, Imaging studies, Environmental sciences, Environmental chemistry, Environmental impact, Chemistry, Analytical chemistry, Environmental chemistry, Characterization and analytical techniques, Imaging techniques, Mass spectrometry

## Abstract

Polyethylene glycols (PEGs) are used in industrial, medical, health care, and personal care applications. The cycling and disposal of synthetic polymers like PEGs pose significant environmental concerns. Detecting and monitoring PEGs in the real world calls for immediate attention. This study unveils the efficacy of time-of-flight secondary ion mass spectrometry (ToF-SIMS) as a reliable approach for precise analysis and identification of reference PEGs and PEGs used in cosmetic products. By comparing SIMS spectra, we show remarkable sensitivity in pinpointing distinctive ion peaks inherent to various PEG compounds. Moreover, the employment of principal component analysis effectively discriminates compositions among different samples. Notably, the application of SIMS two-dimensional image analysis visually portrays the spatial distribution of various PEGs as reference materials. The same is observed in authentic cosmetic products. The application of ToF-SIMS underscores its potential in distinguishing PEGs within intricate environmental context. ToF-SIMS provides an effective solution to studying emerging environmental challenges, offering straightforward sample preparation and superior detection of synthetic organics in mass spectral analysis. These features show that SIMS can serve as a promising alternative for evaluation and assessment of PEGs in terms of the source, emission, and transport of anthropogenic organics.

## Introduction

Polyethylene glycols (PEGs), also known as macrogols, are a versatile family of linear polymers that comprised of repeated oxyethylene (–CH_2_–CH_2_–O–) subunits^1,2^. The molecular formulae are HO(CH_2_CH_2_O)_n_, where n denotes the average number of oxyethylene subunits. This average value is typically greater than or equal to four, signifying the presence of multiple subunits within the polymer chain. Each PEG is followed by a number, e.g., PEG 200, which refers to the average molecular weight of polymer. PEGs encompass a broad range of molecular weights, spanning from 200 to 40,000,000 g/mol. The physical states of PEGs are influenced by their molecular weight of polymers. For instance, PEGs with molecular weights ranging from 200 to 600 g/mol are typically in a liquid state at room temperature. Conversely, PEGs of molecular weights exceeding 1000 g/mol exist as solids, exhibiting a wax-like, paraffin appearance, or as dry powders^1^. These materials exhibit a high solubility in water as well as in a diverse array of organic solvents, including alcohols, esters, ketones, aromatic solvents, and chlorinated solvents^3,4^. This exceptional solubility profile makes PEGs suitable for various applications across multiple fields^5–7^.

At present, PEGs are widespread and used in pharmaceuticals, cosmetics, food, personal care products, and industrial processes^8^. Their solubility characteristics, biocompatibility, non-toxic, and ability to act as dispersing agents, emulsifiers, and stabilizers make them ideal for drug delivery systems, skin creams, and numerous other applications^5,9,10^. PEGs also play a crucial role in the synthesis of nanoparticles, polymerization reactions, and as cryoprotectants^11^. Every year, millions of tons of PEGs are manufactured worldwide and most of them reach conventional sewage disposal systems after industrial utilization^12^. As a result, PEGs are commonly found in industrial and domestic wastewaters, from which they can easily migrate to the aquatic environment^13^. PEG 550 were found in the range of 0.5–68 mg/L in river water and seawater^14^. As PEG and its derivatives are expected to increase their presence in urban and industrial wastewater^15^, concerns have been raised about the fate of these polymers once dispersed in the environment. What’s more, PEGs can be used in tanniniferous forage. As a result, PEG–tannin complex can be detected from the animal feces, demonstrating that the PEG–tannin complex can end up in soil and raise an environment concern^16,17^.

The biological degradation of PEGs is slow, and they stay a long time in the natural environment. This may raise potential subsequent interactions in the human body^18^. Therefore, studies on PEGs are extremely important to understand the distribution, transformation, and removal of these pollutants from the natural environment. Liquid chromatography–mass spectrometry (LC–MS) including reversed-phase LC–MS, normal or reversed-phase high performance liquid chromatography (HPLC), gas chromatography (GC), nuclear magnetic resonance (NMR) spectroscopy, matrix-assisted laser-desorption (MALDI) time-of-flight (TOF) MS, gas–liquid-chromatographic (GLC), and direct injection electrospray-selected ion monitoring mass spectrometry have been used to identify and quantify PEGs in previous studies^19–23^. However, these methods need complex pretreatment procedure to extract or transfer PEGs as derivatives before analysis. Therefore, analysis approaches that offer easy sample preparation and accurate detection are attractive to the community of PEGs research and assess the impacts on the environment, air, water, and human health.

Time-of-flight secondary ion mass spectrometry (ToF-SIMS) is a powerful tool in applied surface analysis. It provides detailed elemental, molecular, and isotopic mass spectra, and two-dimensional/three-dimensional (2D/3D) mapping of solid surfaces, thin films, or gas–solid interfaces^24–34^. Although ToF-SIMS has been used often in biological applications and the semiconductor industry in past decades, its applications are not limited to these well-known fields. A few papers reported that ToF-SIMS was applied for PEG analysis, such as PEG 600, PEG 800, PEG 1000 and PEG 100,000, and a series of characteristic peaks, such as [PEG_*x*_ + H]^+^ and [PEG_*x*_ + Na]^+^ were identified^35–39^. However, only limited PEGs are analyzed and the analysis of real-world samples is absent^40^. ToF-SIMS provides simple and fast sample preparation compared to laborious liquid extraction, for example. It can be used to analyze and identify organics with the limits of detection (LODs) at equivalent of parts per trillion (ppt) level in real environmental samples^41^. ToF-SIMS can produce mass spectra of intact oligomer ions, allowing the calculation of average molecular weights from the moments of the ion peak intensity distribution^36,37^. ToF-SIMS mass spectra provide not only average molecular weight data from intact oligomers but also significant chemical structure information from the various fragments. As a static SIMS analysis technique, ToF-SIMS is semi-quantitative; however, it can provide relative concentrations if the secondary fragments are correctly selected^42^. In addition, as an imaging mass spectrometry technique, it has high mass accuracy and high mass resolving power. Cheminformatics approaches are long established in analytical chemistry (e.g., NMR, Fourier transform ion cyclotron resonance mass spectrometry (FT-ICR-MS), high resolution mass spectrometry, and MS–MS) to determine unknowns in complex mixtures from known chemical information, where observations indicative of chemical structural information are used^43–46^. Similarly, ToF-SIMS spectral results can be used to identify unknowns from known peak information based on observations of signatures of functional groups or characteristic fragments of PEGs. In this work, we demonstrate that ToF-SIMS is a powerful tool for rapid analysis and identification of PEGs. We show that ToF-SIMS can detect and differentiate PEGs with different average molecular weights. These new results enrich the investigation of PEGs. Moreover, we challenged ToF-SIMS in the analysis of real-world cosmetic products. Our results based on spectral analysis, principal component analysis (PCA), and 2D image comparisons show that ToF-SIMS is valid for studying PEGs. Overall, ToF-SIMS can provide more insights and offer an alternative solution to the study of the potential risks associated with PEGs in the natural environment including water, air, or soil.

## Results and discussion

### ToF-SIMS spectral reproducibility of PEG measurements

Previous studies have consistently demonstrated the effectiveness of ToF-SIMS in providing high mass accuracy and reliable spectra reproducibility^24,27,28,47–49^. The LODs of organics have been reported at ppt level in real field collected environmental samples^41^. In this study, seven samples were analyzed (Table S1) and PEG 300 was selected as a representative example to show the reproducibility of static ToF-SIMS spectra. Similar results were obtained for all samples analyzed in this work. The comparison of ToF-SIMS spectra of PEG 300 is presented in Figs. S1a–1c and Figs. S2a–c in the positive ion mode in the *m/z*^+^ range of 0 to 800 in normalized and absolute intensities, respectively. The spectra labeled as P1, P2, P3, P4, P5, and P6 correspond to six sequential measurements of the sample on a silicon (Si) wafer in the positive ion mode. Notably, the ToF-SIMS spectral comparison of PEG 300 demonstrates consistent and reproducible data, where characteristic peaks reappear in all six positive spectra with similar intensities. Also, the spectral comparison in the negative ion mode (Figs. S3–S4) yield similar observations to those in the positive mode.

To quantitatively assess the reproducibility of the SIMS spectra, we performed a statistical analysis of relative mass accuracy of the peak area and peak height using PEG 300 as an example with multiple measurements. It is worth noting that more replicates could increase the estimate of average and standard deviation (S.D.) values based on replication statistics^50^. We generally acquired six to seven data points for each sample in static ToF-SIMS spectral analysis. The benefits to improve repeatability become insignificant after four or five replicates in measurements^51^. When the data points show good measurement precision, we used three replicate measurements to calculate S.D. and average of peaks of each sample for simplicity^41,52^. The characteristic peaks selected for the measurement repeatability evaluation in the positive and negative ion mode were summarized in Tables 1 and S2, respectively. The summary of peak area and peak height ratios of representative peaks in the positive mode is listed in Table S3. The results show that peak area and peak height ratios of key peaks have good measurement precision. The ratios of peak areas and peak heights of key peaks are listed in Table S4. These ratios were calculated using the counts of the specific peak divided by total counts of all selected key products and fragments.

**Table 1:** Possible identification of key peaks of PEGs in the positive ion mode of ToF-SIMS analysis.

*m/z*^+^_theo._^a^	*m/z*^+^_obs._^b^	ΔM^c^, ppm	Formula	Description	References
31.018	31.019	32.239	CH_3_O^+^	Fragment	^58^
45.033	45.035	44.412	C_2_H_5_O^+^	Fragment	^38^
53.002	53.001	18.867	C_3_HO^+^	Fragment	^59^
63.044	63.045	15.862	C_2_H_7_O_2_^+^	HO(CH_2_CH_2_O)H_2_^+^	^38^
81.070	81.074	49.340	C_6_H_9_^+^	Fragment	^27^
89.060	89.064	44.914	C_4_H_9_O_2_^+^	Fragment	^35,60^
107.070	107.075	46.698	C_4_H_11_O_3_^+^	HO(CH_2_CH_2_O)_2_H_2_^+^	^38^
129.052	129.059	54.242	C_4_H_10_O_3_Na^+^	HO(CH_2_CH_2_O)_2_HNa^+^	^38^
133.086	133.094	60.112	C_6_H_13_O_3_^+^	Fragment	^35^
151.096	151.098	13.237	C_6_H_15_O_4_^+^	Fragment	^35^
173.078	173.082	23.111	C_6_H_14_O_4_Na^+^	HO(CH_2_CH_2_O)_3_HNa^+^	This study
175.096	175.103	39.978	C_8_H_15_O_4_^+^	Fragment	^35^
177.112	177.117	28.231	C_8_H_17_O_4_^+^	Fragment	^35^
195.123	195.124	5.125	C_8_H_19_O_5_^+^	HO(CH_2_CH_2_O)_4_H_2_^+^	^38^
217.105	217.106	4.606	C_8_H_18_O_5_Na^+^	HO(CH_2_CH_2_O)_4_HNa^+^	This study
221.154	221.150	18.087	C_14_H_21_O_2_^+^	Fragment	^61^
239.149	239.148	4.181	C_10_H_23_O_6_^+^	HO(CH_2_CH_2_O)_5_H_2_^+^	^38^
261.131	261.128	11.488	C_10_H_22_O_6_Na^+^	HO(CH_2_CH_2_O)_5_HNa^+^	This study
283.175	283.176	3.531	C_12_H_27_O_7_^+^	HO(CH_2_CH_2_O)_6_H_2_^+^	^38^
305.158	305.151	22.939	C_12_H_26_O_7_Na^+^	HO(CH_2_CH_2_O)_6_HNa^+^	This study
327.201	327.202	3.056	C_14_H_31_O_8_^+^	HO(CH_2_CH_2_O)_7_H_2_^+^	^38^
349.183	349.181	5.728	C_14_H_30_O_8_Na^+^	HO(CH_2_CH_2_O)_7_HNa^+^	This study
371.228	371.227	2.694	C_16_H_35_O_9_^+^	HO(CH_2_CH_2_O)_8_H_2_^+^	^38^
393.210	393.206	10.173	C_16_H_34_O_9_Na^+^	HO(CH_2_CH_2_O)_8_HNa^+^	This study
415.254	415.252	4.816	C_18_H_39_O_10_^+^	HO(CH_2_CH_2_O)_9_H_2_^+^	^35,38^
437.236	437.237	2.287	C_18_H_38_O_10_Na^+^	HO(CH_2_CH_2_O)_9_HNa^+^	This study
459.280	459.279	2.177	C_20_H_43_O_11_^+^	HO(CH_2_CH_2_O)_10_H_2_^+^	^35,38^
481.262	481.263	2.078	C_20_H_42_O_11_Na^+^	HO(CH_2_CH_2_O)_10_HNa^+^	^35^
503.306	503.307	1.987	C_22_H_47_O_12_^+^	HO(CH_2_CH_2_O)_11_H_2_^+^	^35,38^
525.288	525.293	9.519	C_22_H_46_O_12_Na^+^	HO(CH_2_CH_2_O)_11_HNa^+^	^35^
547.332	547.300	58.465	C_24_H_51_O_13_^+^	HO(CH_2_CH_2_O)_12_H_2_^+^	^38^
569.314	569.281	57.964	C_24_H_50_O_13_Na^+^	HO(CH_2_CH_2_O)_12_HNa^+^	^35^
591.359	591.354	8.455	C_26_H_55_O_14_^+^	HO(CH_2_CH_2_O)_13_H_2_^+^	^35,38^
613.341	613.352	17.935	C_26_H_54_O_14_Na^+^	HO(CH_2_CH_2_O)_13_HNa^+^	^35^
635.385	635.399	22.034	C_28_H_59_O_15_^+^	HO(CH_2_CH_2_O)_14_H_2_^+^	^35,38^
657.367	657.370	4.564	C_28_H_58_O_15_Na^+^	HO(CH_2_CH_2_O)_14_HNa^+^	^35^
679.411	679.417	8.831	C_30_H_63_O_16_^+^	HO(CH_2_CH_2_O)_15_H_2_^+^	^35,38^
701.393	701.392	1.426	C_30_H_62_O_16_Na^+^	HO(CH_2_CH_2_O)_15_HNa^+^	^35^

^a^*m/z*^+^_theo_: theoretical mass to charge ratio in the positive ion mode.

^b^*m/z*^+^_obs._: observed mass to charge ratio in the positive ion mode.

^c^ΔM: = Abs (10^6^ × (*m/z*^−^_obs._ − *m/z*^−^_theo._)/ *m/z*^−^_theo._) (expressed in ppm)^41,54^.

The relative standard deviations percentage (RSD%) of peak area ranges from 0.90 to 6.47%, with an average of 3.40%. As to peak area ratios, the RSDs% range from 0.00 to 9.09% with an average of 2.32%. It has been reported if the RSD% is 5% in peak areas, the method would be considered suitable for quantitative analysis^53^. Our results show that most RSD% values of the peak area fall below 5%, underscoring the robust reproducibility of the static SIMS spectral measurements. When considering the peak height, the RSD% ranges between 0.81 and 12.13% with an average of 4.24%, slightly larger than the peak area calculation. Similar results are obtained for the peak height ratios. Overall, our results indicate that the SIMS spectral measurements have excellent reproducibility in the analysis of pure PEG and HEG reference samples.

Similarly, good SIMS spectral reproducibility is obtained in the negative ion mode (Figs. S3a–c and Figs. S4a–c). The RSDs% of peak area range from 0.24 to 8.23% with an average value of 3.61% (Table S5). The RSD% peak area ratios are between 1.11 and 5.86% with an average of 3.11% (Table S6). Our result shows that most RSDs% of the peak area fall below 5% (Table S5), indicating excellent spectral reproducibility in the negative ion mode. Additionally, the signal to noise ratio (SNR) of the peaks selected for the evaluation of measurement repeatability are all much bigger than three, indicating that the peaks are signal not noise (Tables S7–S8). More information and detailed statistical analysis results are provided in the supporting information.

### PEG characteristic peaks reveled in ToF-SIMS

**Figure 1:**
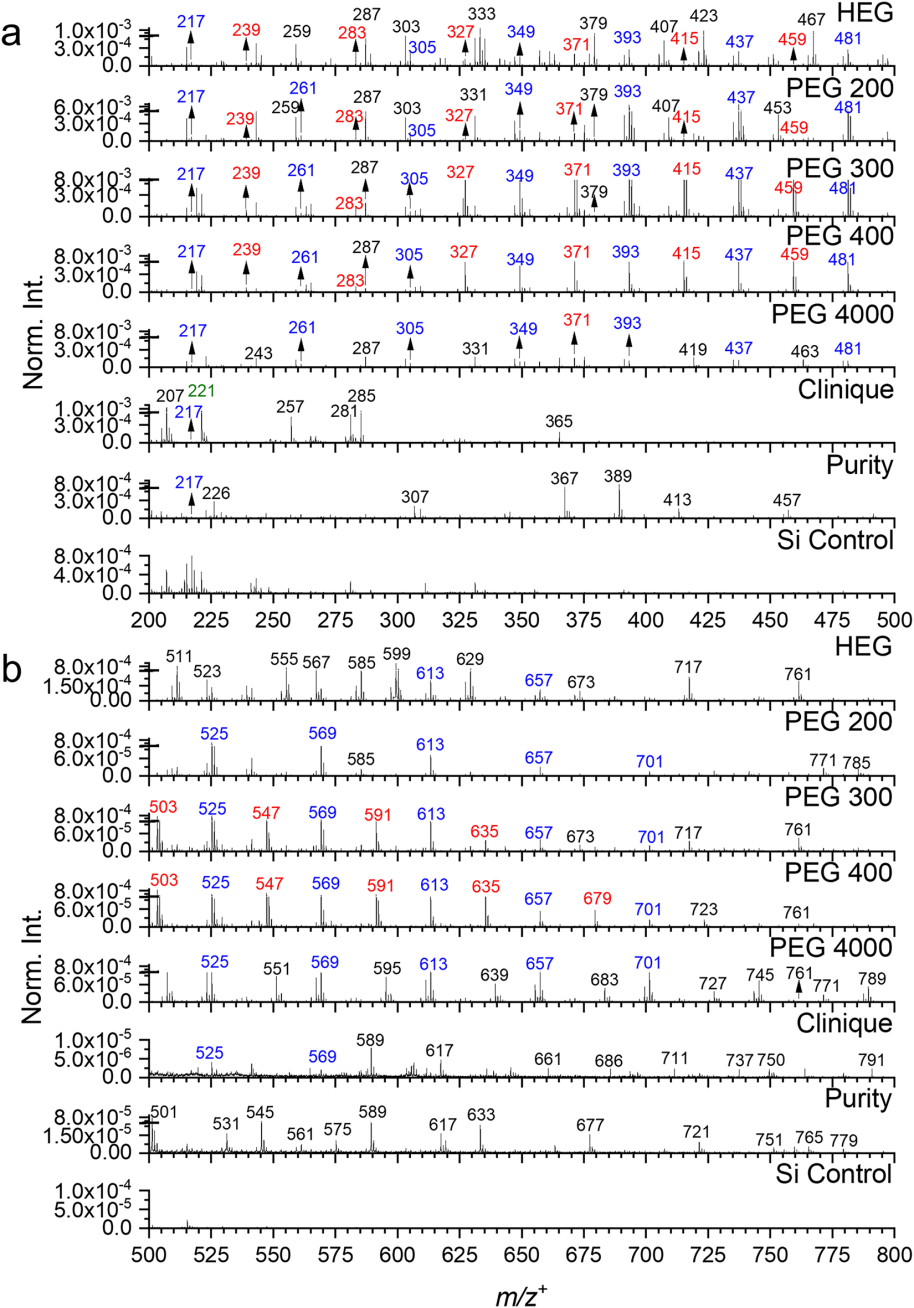
ToF-SIMS spectral comparison of HEG, PEGs, Clinique, and Purity: (**a**) *m/z*^+^ 200–500 and (**b**) *m/z*^+^ 500–800. Normalized intensity (Norm. Int.) is calculated using total ion intensities. The red, blue, and green color represent different series of PEG peaks, namely red stands for HO(CH_2_CH_2_O)_n_H_2_^+^, blue HO(CH_2_CH_2_O)_n_HNa^+^, and green fragment peaks.

Tables 1 and S2 summarize the identified peaks in the positive mode. Figure 1 depicts ToF-SIMS spectral comparison of HEG, PEG 200, PEG 300, PEG 400, PEG 4000, Clinique, Purity, and Si substrate control in the positive ion mode, in the *m/z*^+^ range of 200 to 800. Figure S5 shows comparison of *m/z*^+^ 0–200, while additional negative spectral compassions are depicted in Figs. S6a–c. ToF-SIMS is often regarded as a semi-quantitative technique^54^. In order to compare the relative abundance of peaks in the series of PEG and HEG reference samples, normalized intensities are used. Also, the spectral comparisons in absolute counts are shown in Figs. S7–S8. The absolute spectral comparisons show that the counts of the peaks discussed in this study are reasonable, and SNR are generally several tens of thousands.

A series of PEG characteristic peaks (Table 1) are observed with good intensities and the counts of those peaks are in the range of a few hundreds to several hundreds of thousands for the following peaks, such as *m/z*^+^ 63.045 C_2_H_7_O_2_^+^, 107.075 C_4_H_11_O_3_^+^, 151.098 C_6_H_15_O_4_^+^, 195.123 C_8_H_19_O_5_^+^, 239.148 C_10_H_23_O_6_^+^, 283.176 C_12_H_27_O_7_^+^, 327.201 C_14_H_31_O_8_^+^, 371.227 C_16_H_35_O_9_^+^, 415.252 C_18_H_39_O_10_^+^, 459.279 C_20_H_43_O_11_^+^, 503.307 C_22_H_47_O_12_^+^, 547.300 C_24_H_51_O_13_^+^, 591.354 C_26_H_55_O_14_^+^, 635.399 C_28_H_59_O_15_^+^, and 679.417 C_30_H_63_O_16_^+^. These peaks show a regular mass increase, and they can be expressed by the general formula, i.e., HO(CH_2_CH_2_O)_n_H_2_^+^, where n ranges from 1 to 15 (Figs. 1 and S5, labeled in red color). These peaks are formed when the PEG molecules HO(CH_2_CH_2_O)_n_H get a H atom. The observation of the series peaks indicates that ToF-SIMS can capture the characteristic peak patterns in PEG samples. Among these identified characteristic peaks, *m/z*^+^ 63.045 C_2_H_7_O_2_^+^ is observed with a high relative intensity in HEG and Purity samples, but lower intensities in Clinique and other PEG samples (Fig. S5). The peak *m/z*^+^ 503.307 C_22_H_47_O_12_^+^ is observed with high intensities in PEG 300 and PEG 400 samples; and the peak *m/z*^+^ 679.417 C_30_H_63_O_16_^+^ is observed with high intensities only in the PEG 400 sample (Fig. 1b). The above observations provide the evidence that ToF-SIMS can identify differences in peak intensities among the samples. Additionally, the PEG characteristic peak can be detected in real-world cosmetic products (i.e., Clinique and Purity samples) using ToF-SIMS. The PEGs fragment *m/z*^+^ 31.019 CH_3_O^+^ identified in Clinique and Purity also supports the latter finding (Fig. S5). The peaks *m/z*^+^ 327.201 C_14_H_31_O_8_^+^, 371.227 C_16_H_35_O_9_^+^, 415.252 C_18_H_39_O_10_^+^, 459.279 C_20_H_43_O_11_^+^, 547.300 C_24_H_51_O_13_^+^, 591.354 C_26_H_55_O_14_^+^ and 635.399 C_28_H_59_O_15_^+^ are observed with high intensities in PEG 300 and PEG 400 samples (Fig. 1a,b), indicating that PEG 300 and 400 samples share some similar properties to certain extent. In addition, the peaks *m/z*^+^ 151.098 C_6_H_15_O_4_^+^, 195.123 C_8_H_19_O_5_^+^, 239.148 C_10_H_23_O_6_^+^, and 283.176 C_12_H_27_O_7_^+^ are observed with low intensities in HEG and PEG samples (Figs. S5 and 1a). This observation suggests that the HEG share some common peaks with PEGs. Furthermore, the peak *m/z*^+^ 195.123 C_8_H_19_O_5_^+^ is observed in Clinique and Purity samples, providing additional support for the identification of PEG characteristic peaks in real-world cosmetic products using ToF-SIMS.

Another series of PEG characteristic peaks, which can be expressed as HO(CH_2_CH_2_O)_n_HNa^+^ with n ranging from 2 to 15, is observed in the samples with varying intensities (Figs. 1 and S5, labeled in bule color). It is well known that Na^+^ can be easily added on polymer fragment ions to enhance ionization yield in positive ion ToF-SIMS spectra^35^. Among these peaks, *m/z*^+^ 129.059 C_4_H_10_O_3_Na^+^ is observed not only in HEG and PEG samples but also in Clinique and Purity samples (Fig. S5). This observation proves that ToF-SIMS can detect PEGs in real-world cosmetic products. The peaks *m/z*^+^ 261.128 C_10_H_22_O_6_Na^+^ and 305.151 C_12_H_26_O_7_Na^+^ are observed with low intensities in HEG and all PEG samples (Fig. 1a). The peaks *m/z*^+^ 349.181 C_14_H_30_O_8_Na^+^ is observed with relatively high intensities in PEG 300 and PEG 400 samples but lower intensities in HEG and PEG 4000 samples (Fig. 1a). The two observations suggest HEG and PEGs not only share some common properties but also exhibit significant differences. ToF-SIMS can detect both the common and distinct characteristic peaks. For example, the peaks *m/z*^+^ 393.206 C_16_H_34_O_9_Na^+^, 437.237 C_18_H_38_O_10_Na^+^, and 481.263 C_20_H_42_O_11_Na^+^ exhibit higher intensities in PEG 200, PEG 300, and PEG 400 samples compared to HEG and PEG 4000 samples (Fig. 1a), indicating the chemical composition of PEG 200, PEG 300 and PEG 400 are similar.

Additionally, PEG 4000 shares some common peaks with PEG 200, PEG 300, and PEG 400, exhibiting high intensities. These peaks are identified as *m/z*^+^ 525.293 C_22_H_46_O_12_Na^+^, 569.281 C_24_H_50_O_13_Na^+^, and 613.352 C_26_H_54_O_14_Na^+^ (Fig. 1b). The peak *m/z*^+^ 569.281 C_24_H_50_O_13_Na^+^ is also observed in Clinique, further indicating that ToF-SIMS can detect PEG peaks in the real-world cosmetic products. Peaks, such as *m/z*^+^ 657.370 C_28_H_58_O_15_Na^+^ and 701.392 C_30_H_62_O_16_Na^+^, are observed with highest intensities in PEG 40,000, but lower intensities in PEG 200, PEG 300, and PEG 400 samples (Fig. 1b). These two observations confirm that the series of PEG samples not only share common peaks but also bear significant differences. All the observations collectively support the conclusion that ToF-SIMS is a powerful tool for capturing characteristic peak patterns and identifying differences among PEG samples.

Apart from the two series of PEG characteristic peaks, several fragment peaks of PEGs are identified in samples using static ToF-SIMS. The peaks include but not limit to *m/z*^+^ 31.019 CH_3_O^+^, 45.035 C_2_H_5_O^+^, 53.001 C_3_HO^+^, 81.074 C_6_H_9_^+^, 89.064 C_4_H_9_O_2_^+^, 133.094 C_6_H_13_O_3_^+^, 175.103 C_8_H_15_O_4_^+^ and 221.150 C_14_H_21_O_2_^+^ (Figs. S5 and 1a, labeled with green color). Among these peaks, *m/z*^+^ 31.019 CH_3_O^+^is observed with high intensities in HEG and all PEG samples, but lower intensities in Purity and Clinique samples. The peak *m/z*^+^ 53.001 C_3_HO^+^ is observed with relative high intensities not only in HEG and PEG 200 samples but also in Purity. In addition, peak *m/z*^+^ 133.094 C_6_H_13_O_3_^+^ is observed with high intensities in Clinique and PEG 300, PEG 400, and PEG 4000. However, the peak *m/z*^+^ 81.074 C_6_H_9_^+^ is observed with high intensities only in HEG and Purity. These observations provide solid evidence that ToF-SIMS detects PEGs in real-world cosmetic products.

The ToF-SIMS spectral comparison of the seven samples and the Si substrate control in the negative ion mode show similar results to those in the positive ion mode (Figs. S6a–c and S9a–c). The consistent spectral observations in both positive and negative ion modes imply that ToF-SIMS can capture characteristic peak patterns of PEG samples and effectively identify differences among various PEG samples. Moreover, our results emphasize the significance of ToF-SIMS as a viable and reliable approach for studying PEGs present in real-world cosmetic products. Its ability to detect and differentiate PEGs in complex mixtures, such as cosmetic formulations like Clinique and Purity, further underscores ToF-SIMS’ potential as an essential analytical tool for investigating and assessing the presence of PEGs in various real-world applications in environment.

### The spectral signatures in PEGs and real cosmetic samples identified by PCA

PCA, a widely used multivariate analysis technique, was employed to discern differences among the samples in ToF-SIMS analysis^24,27,28,33^. The scores plots and loadings plots are typically presented together to analyze the variances within the samples. Scores plots depict the similarity and dissimilarity among samples, while loadings plots provide insights into the contributions of the components corresponding to the principal components' (PC) scores. In this study, selected peak spectral PCA was conducted to validate the spectral observation and to identify additional features among the series of samples and their components.

**Figure 2:**
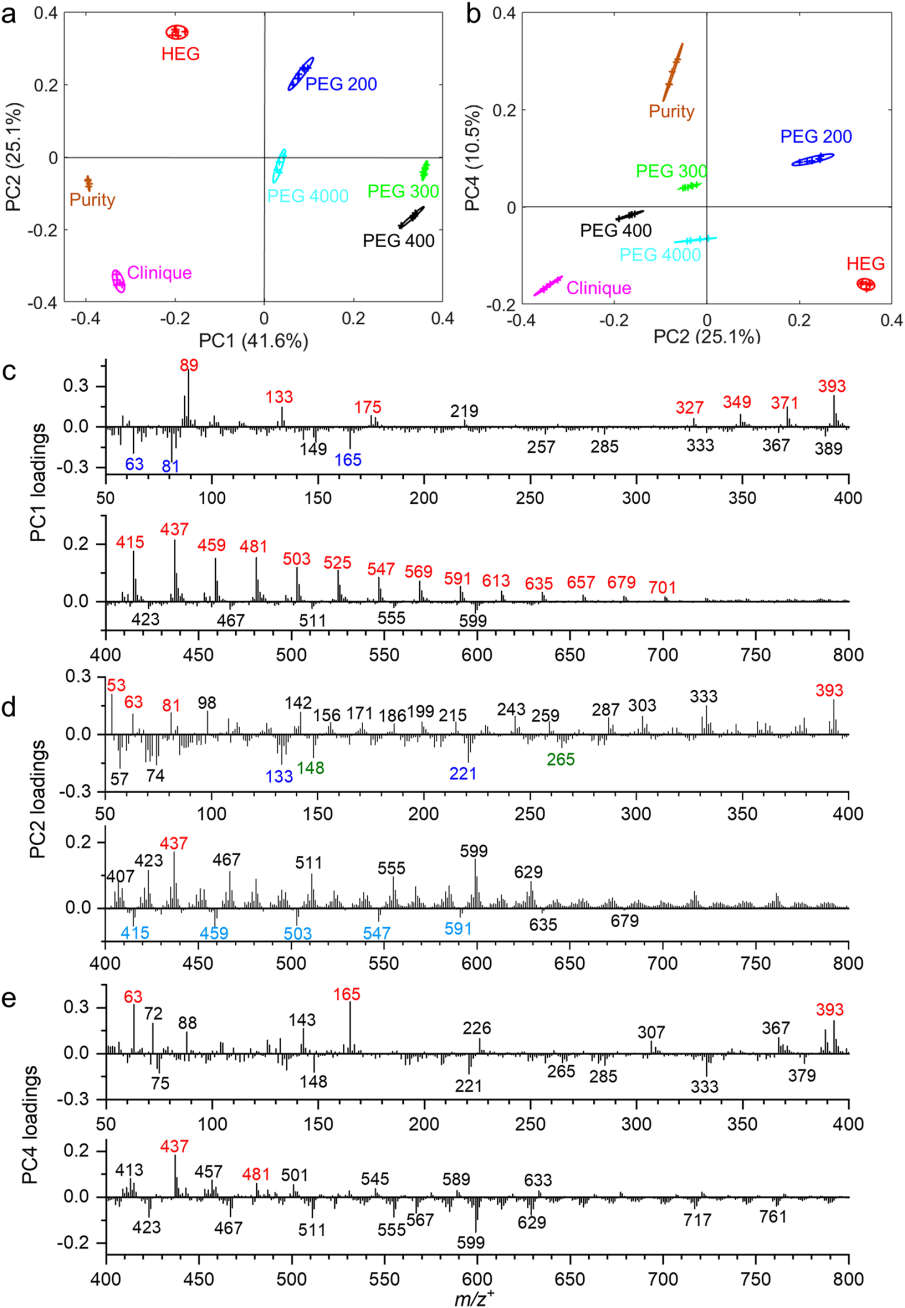
ToF-SIMS spectral PCA results of HEG, PEGs, Clinique, and Purity in the positive mode: Scores plots of PC1 vs. PC2 (**a**), PC2 vs. PC4 (**b**), PC1 (**c**), PC2 (**d**), and PC4 (**e**) loadings plots in *m/z*^+^ 50–800. Peaks are labelled in their center masses. The red and dark blue (or light blue) colors are used to mark contributing peaks in the positive and negative PC loadings, respectively. The green color represents peaks that could not be unidentified.

Figure 2 displays PC1 versus (vs.) PC2, PC2 vs. PC4, and their loadings plots in the positive mode. Figure S10 shows PC2 vs. PC3 and PC3 loading plots. PC1, PC2, PC3 and PC4 can explain more than 90% of all data. Specifically, PC1 explains 41.6% of data and primarily separates PEG 200, PEG 300, and PEG 400 from HEG, Purity, and Clinique. PC2 explains 25.1% of data and separates HEG and PEG 200 from PEG 300, PEG 400, PEG 4000, Purity, and Clinique. PC3 explains 13.6% of data and separates PEG 200, PEG 300, and Clinique from HEG, PEG 400, PEG 400 and Clinique. PC4 explains 10.5% of data and separates PEG 200, PEG 200, and Purity from HEG, PEG 400, and Clinique. In PC1 positive mode loadings (Fig. 2c), PEGs including PEG 200, PEG 300, PEG 400, and PEG 4000 are the main contributors, indicating that the series of PEG samples share significant similarities in their components. It’s not surprising to observe similar signatures in PEG samples, considering that they all possess the same chemical formula of HO(CH_2_CH_2_O)_n_H. The characteristic peaks, such as *m/z*^+^ 89.064 C_4_H_9_O_2_^+^, 133.094 C_6_H_13_O_3_^+^, 175.103 C_8_H_15_O_4_^+^, 327.201 C_14_H_31_O_8_^+^, 349.181 C_14_H_30_O_8_Na^+^, 371.227 C_16_H_35_O_9_^+^, 393.206 C_16_H_34_O_9_Na^+^, 415.252 C_18_H_39_O_10_^+^, 437.237 C_18_H_38_O_10_Na^+^, 459.279 C_20_H_43_O_11_^+^, 481.263 C_20_H_42_O_11_Na^+^, 503.307 C_22_H_47_O_12_^+^, 525.293 C_22_H_46_O_12_Na^+^, 547.300 C_24_H_51_O_13_^+^, 569.281 C_24_H_50_O_13_Na^+^, 591.354 C_26_H_55_O_14_^+^, 613.352 C_26_H_54_O_14_Na^+^, 635.399 C_28_H_59_O_15_^+^, 657.370 C_28_H_58_O_15_Na^+^, 679.417 C_30_H_63_O_16_^+^, and 701.392 C_30_H_62_O_16_Na^+^, make great contributions to the variance (Fig. 2c, labeled in red color). PC1 negative separates HEG, Purity, and Clinique from the series of PEG samples (Fig. 2a). This finding suggests that the composition of the three samples is largely different from the series of PEG samples. In PC1 negative mode loadings, fewer organic fragments (Fig. 2c, labeled with dark blue color), such as *m/z*^+^ 63.045 CH_3_O^+^, 81.074 C_6_H_9_^+^, and 165.119 (not identified), display high loadings, showing that these peaks contribute more significantly in HEG, Purity, and Clinique.

The PC2 negative scores in Fig. 2a reveals that PEG 300, PEG 400, PEG 4000, Purity, and Clinique share the same peaks. This finding provides further confirmation that characteristic PEG peaks are indeed detected in real-world cosmetic products. In PC2 negative loadings, peaks such as *m/z*^+^ 133.094 C_6_H_13_O_3_^+^ and 221.150 C_15_H_25_O^+^ (Fig. 2d, labeled in dark blue color) contribute significantly more to the variance compared to peaks such as *m/z*^+^ 415.252 C_18_H_39_O_10_^+^, 459.279 C_20_H_43_O_11_^+^, 503.307 C_22_H_47_O_12_^+^, 547.300 C_24_H_51_O_13_^+^, 591.354 C_26_H_55_O_14_^+^ (labeled in light blue), and several unidentified peaks, including *m/z*^+^ 148.077 and 265.170 (Fig. 2d, labeled in green color). The PC2 positive scores demonstrate that HEG shares common peaks with PEG 200 (Fig. 2a). This observation is reasonable because HEG and PEG 200 both contain the chemical compound C_12_H_26_O_7_, which can form similar fragments when analyzed by ToF-SIMS. The peaks, including but not limited to *m/z*^+^ 53.001 C_3_HO^+^, 63.045 C_2_H_7_O_2_^+^, 81.074 C_6_H_9_^+^, 393.206 C_16_H_34_O_9_Na^+^, and 437.237 C_18_H_38_O_10_Na^+^, play a more significant role in distinguishing HEG and PEG 200 from the other samples (Fig. 2d, labeled in red color). The PC3 positive scores in Fig. S10a reveals that PEG 200, PEG 300, and Clinique share same peaks. The peaks, such as 221.150 C_14_H_21_O_2_^+^, 393.206 C_16_H_34_O_9_Na^+^, 437.237 C_18_H_38_O_10_Na^+^, and 481.263 C_20_H_42_O_11_Na^+^ are observed with high loadings in PC3 positive loadings (Fig. S10b). PC3 negative scores separate Purity from Clinique, showing the compositional differences between the two samples. Figure 2b depicts PC2 vs. PC4 scores plot. It is evident that Purity exhibits some components that are also found in PEG 200 and PEG 300, providing further support for the detection of PEG-related peaks in real-world cosmetic products. The peaks, such as *m/z*^+^ 63.045 C_2_H_7_O_2_^+^, 165.119 (not identified), 393.206 C_16_H_34_O_9_Na^+^, 437.237 C_18_H_38_O_10_Na^+^, and 481.263 C_20_H_42_O_11_Na^+^ are representative components in PC4 positive loadings (Fig. 2e, labeled in red color). The PC4 negative scores plot reveals that Clinique shares common peaks with PEG 400, PEG 4000, and HEG. This observation agrees with the previous result, indicating the detection of PEG characteristic peaks in cosmetic products. Furthermore, PC4 negative scores separate Clinique from Purity, suggesting significant compositional differences between these two cosmetic products.

Overall, the SIMS spectral PCA results obtained from the scores and loadings plots provide valuable insights into the shared characteristics and distinctive features among the analyzed samples. The observations support the presence of PEG characteristic peaks in cosmetic products and highlight compositional differences between different product categories. The scores plots of PC1 vs. PC2, PC2 vs. PC4, and their corresponding loadings plots in the negative mode can be found in Fig. S11. These negative mode results agree with the findings from the positive mode plots. Further details and comprehensive information are available in the supporting information.

### Fragments of PEGs in cosmetic products confirmed by SIMS 2D imaging

**Figure 3:**
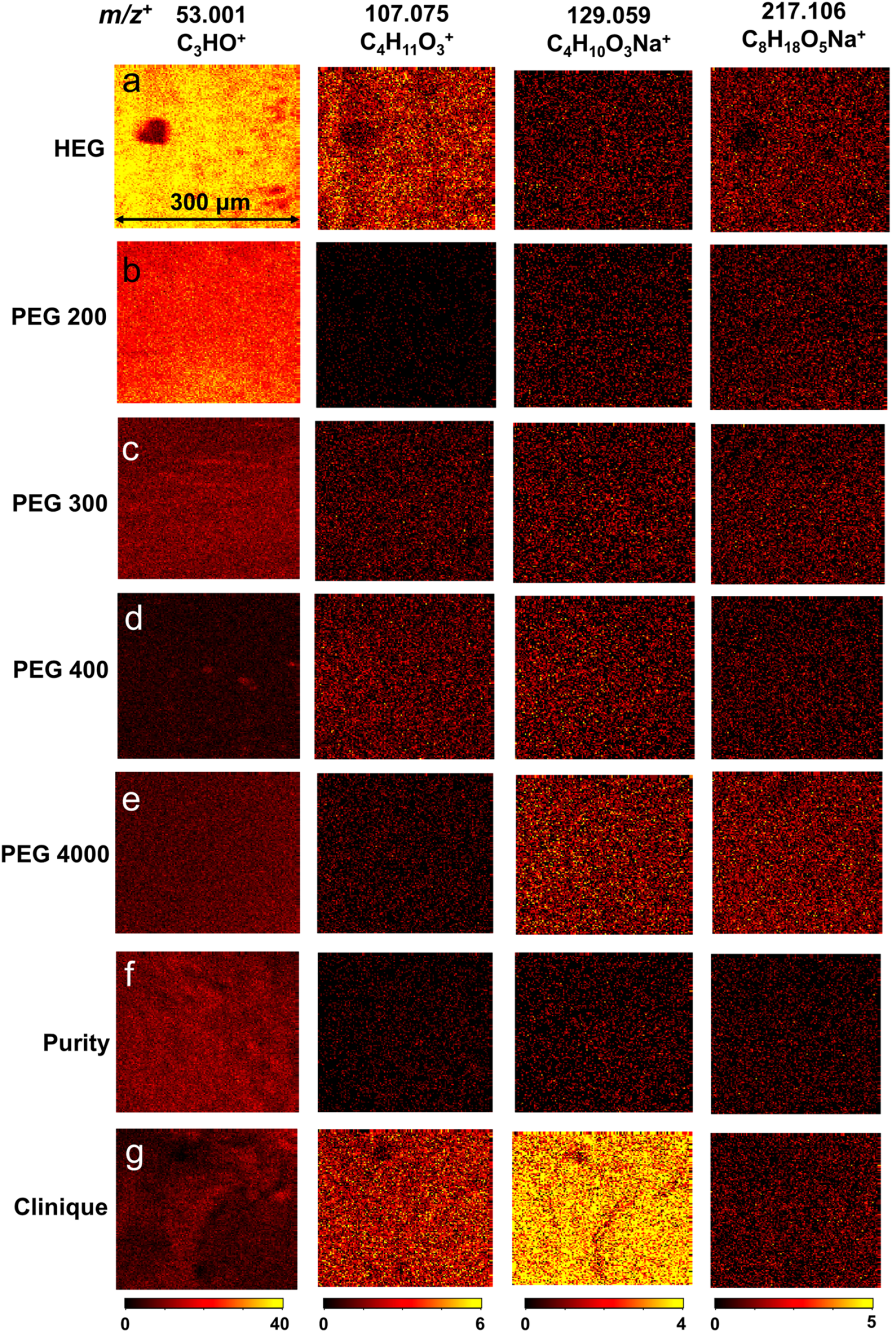
ToF-SIMS 2D image comparison of key peaks in the positive mode: (**a**) HEG sample, (**b**) PEG 200 sample, (**c**) PEG 300 sample, (**d**) PEG 400 sample, (**e**) PEG 4000 sample, (**f**) Purity sample, and (**g**) Clinique sample.

Figures 3 and S12 illustrate the SIMS 2D image comparison of selected key peaks observed in HEG, PEGs, and real-world cosmetic products in the positive ion mode. The color scale ranging from red to dark signifies higher and lower relative ion intensities, respectively. The absolute counts of these peaks are reasonable, and the SNR of these peaks are in the range of 36 to 2360 (Table S7), suggesting the peaks are reliable signals. The same applies to other samples studied in this work. This means that the peaks discussed herein are based on reasonable measurements. Additional normalized 2D images are seen in the supporting information (Figs. S13–S14).

The 2D image comparison results support the spectral observations in the positive mode. First, it confirms that HEG and PEGs share certain common component peaks, albeit with varying intensities, highlighting the similarities and differences among the samples. For instance, peaks such as *m/z*^+^ 129.059 C_4_H_10_O_3_Na^+^ and 217.106 C_8_H_18_O_5_Na^+^ are observed with low intensities in all PEG and HEG samples (Fig. 3). On the other hand, peaks like *m/z*^+^ 371.227 C_16_H_35_O_9_^+^ and 415.252 C_18_H_39_O_10_^+^ are detected with higher intensities in PEG 300 and PEG 400 compared to the other samples (Fig. S12). Notably, the prominent peaks *m/z*^+^ 657.370 C_28_H_58_O_15_Na^+^ and 701.392 C_30_H_62_O_16_Na^+^ are more abundant in PEG 4000 compared to other PEGs (Fig. S12). Secondly, the presence of PEG fragments in real-world cosmetic products is clearly detected using ToF-SIMS. For instance, peaks like *m/z*^+^ 53.001 C_3_HO^+^, 107.075 C_4_H_11_O_3_^+^, 129.059 C_4_H_10_O_3_Na^+^, and 217.106 C_8_H_18_O_5_Na^+^ are detected in Purity and Clinique samples (Fig. 3). However, peaks like *m/z*^+^ 53.001 C_3_HO^+^ and 107.075 C_4_H_11_O_3_^+^ are observed with high intensities in both Purity and Clinique, while the peak *m/z*^+^ 53.001 C_3_HO^+^ is detected with higher intensity in Purity compared to Clinique, and the peaks *m/z*^+^ 107.075 C_4_H_11_O_3_^+^ and 129.059 C_4_H_10_O_3_Na^+^ exhibit higher intensity in Clinique. The distribution of PEGs in complex real-world cosmetic formulations like Purity and Clinique implies that ToF-SIMS has the potential ability to effectively analyze PEGs within samples collected from natural environments.

Additionally, normalized, and absolute 2D image comparisons of selected key peaks in the negative ion mode (Figs. S15–S16) yield similar observations and support the findings obtained in the positive mode. Further details can be found in the supporting information.

## Conclusion

We demonstrate that ToF-SIMS is a powerful tool to analyze PEGs with simple sample preparation. Our results show good mass spectral repeatability in static SIMS measurements in both positive and negative ion mode. Most values of the RSD% of peak area fall below 5%, showing an excellent reproducibility of the SIMS spectral measurements of reference PEGs and HEG. The key peaks and fragments can be identified with relative mass accuracy of less than 65 ppm. The SNRs of representative peak fragments range from several hundreds to tens of thousands, indicating that characteristic peaks of PEGs could be analyzed using ToF-SIMS effectively. The static SIMS spectral comparison results highlight the robustness of ToF-SIMS in capturing characteristic peak patterns and distinguishing variations among samples containing PEGs. The spectral PCA results further confirm the findings in spectral analysis; and multivariate analysis effectively discriminates compositions among different samples. Moreover, we demonstrate that ToF-SIMS can detect PEGs in commercial cosmetic products. Such results provide valuable proof that SIMS is a viable method for studying PEGs in environment. This analytical approach could have a significant impact on quality control, safety assessment, and regulatory compliance in industries dealing with PEG-containing products. Overall, results presented in this SIMS study are attractive for complex organic detection due to its simplicity in sample preparation and efficiency for sample analysis. More standard chemicals and reference samples containing PEG and target products are warranted for analysis in ToF-SIMS to establish a rich reference library of data for its application in environmental organics like PEGs and other complex and challenging pollutant analysis in the future.

## Methods

### Chemicals

A series of samples were prepared in this study, including one 2-(Hexyloxy)ethanol (HEG, C_8_H_18_O_2_), four PEGs, and two real-world cosmetic products. The samples description was summarized in Table S1. The chemicals HEG, PEG 200, PEG 300, and PEG 400 used in this work were acquired from Sigma-Aldrich (St. Louis, MO, USA), and PEG 4000 was acquired from Alfa Aesar (A Johnson Matthey Company). The real-world cosmetic products Purity (one-step facial cleanser, Philosophy, USA) and Clinique (dramatically different moisturizing gel, Clinique, USA) were purchased from Macy’s (a department store in USA). All materials were used as is without further purification.

### ToF-SIMS sample preparation

Prior to sample preparation, Si wafers underwent a sequential cleaning process involving acetone, isopropanol, and high-purity DI water for a duration of 3 min each^24,28^. After thorough cleaning using solvents, a UV-O_3_ plasma treatment (Model No. 342, Jelight Company Inc., USA) was performed for 2 min. to remove remaining organic contaminants on the surface of the Si wafer. Sample preparation was carried out within a fume hood (Hamilton Laboratory Solution LLC., WI, USA).

**Figure 4:**
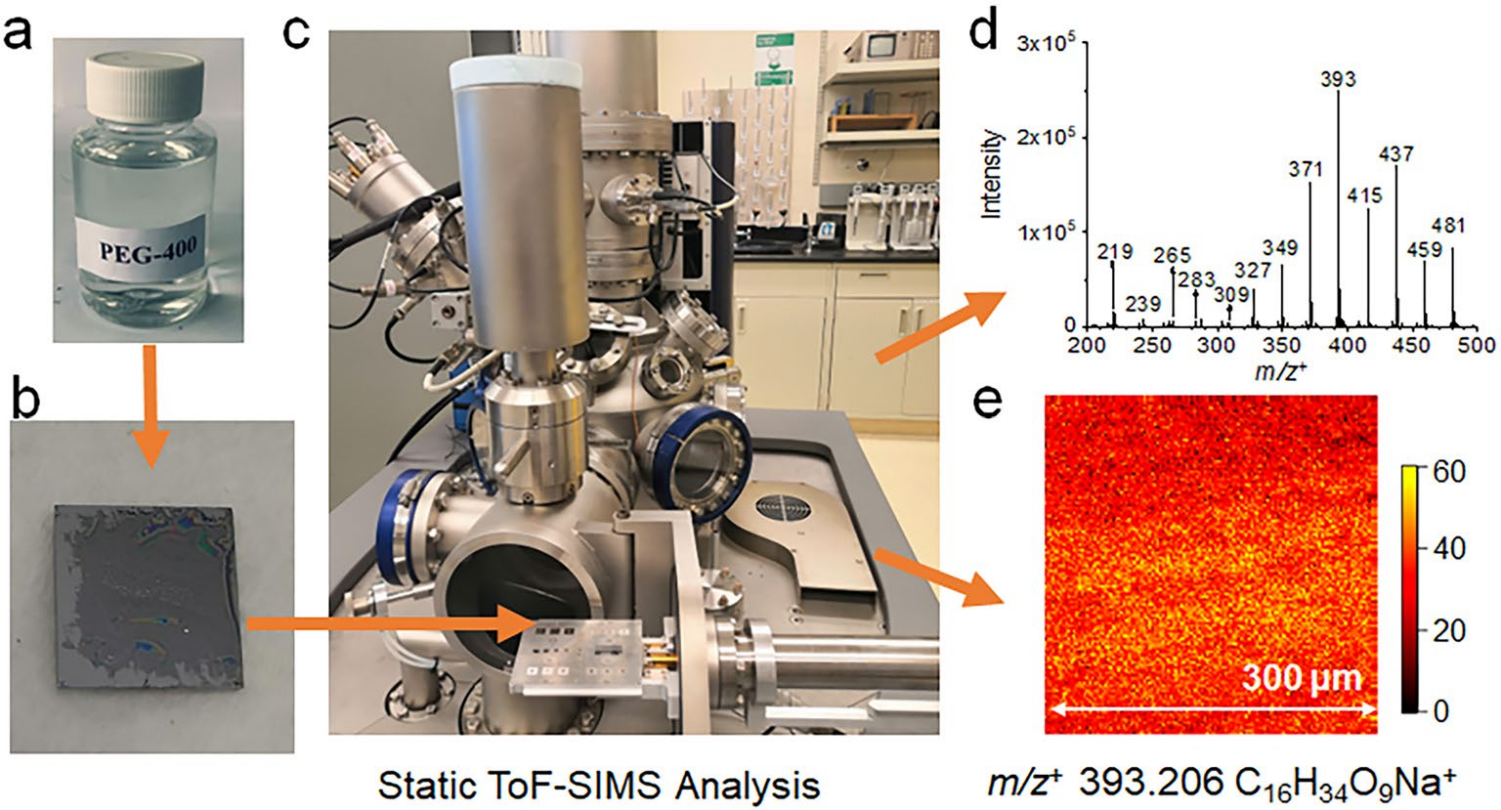
The schematic showing PEG sample preparation and analysis in static ToF-SIMS: (**a**) picture of the PEG 400 material; (**b**) dry PEG 400 film prepared on the Si wafer for ToF-SIMS analysis; (**c**) the ToF-SIMS back mount stage with samples loaded prior to analysis; (**d**) a representative SIMS mass spectrum, and (**e**) a 2D image of *m/z*^+^ 393.206 C_16_H_34_O_9_Na^+^ obtained from the PEG sample.

The schematic of PEG sample preparation and analysis in static ToF-SIMS are illustrated in Fig. 4. Among these chemicals, HEG, PEG 200, PEG 300, PEG 400, Purity, and Clinique exist in a liquid state at room temperature. As to these liquid chemicals, the samples for SIMS analysis were prepared by the following steps: (1) the clean Si wafer (10 × 10 mm^2^ diced, Ted Pella Inc., CA, USA) was put in a new petri dish; (2) then 10 μL of liquid sample was deposited on the cleaned Si wafer using a pipette; (3) another clean Si wafer was used to repeatedly scrape the surface with the droplet until it spread as a thin layer. This is because the PEG or HEG droplet was difficult to dry at its natural state even using nitrogen blowing. On the other hand, PEG 4000 exists in a solid state at room temperature, the sample was prepared by diluting 1.0 g of solid powder in 50 mL DI water (18.2 MΩ) dispensed from a Barnstead water purification system (Nanopure diamond model) to obtain a 20 mg/mL solution. The PEG 4000 sample was then prepared following the steps described above using the solution. The prepared samples on Si substrates were immediately analyzed in ToF-SIMS to avoid possible surface interference and contamination.

### ToF-SIMS analysis

A ToF-SIMS V instrument (IONTOF GmbH, Münster, Germany) was used to analyze samples. During analysis, the pressure in the main chamber was remained at ~ 1 × 10^–8^ mbar, and it took about 0.5 h for the vacuum to the desirable condition in the load lock chamber. A 25 keV Bi_3_^+^ primary ion beam with ~ 450 nm in diameter and 10 kHz pulse energy was used for spectral measurements. The pulse width was 0.8 ns, and the current was set at ~ 0.6 pA. Scanning for SIMS spectra was carried out over an aera of 300 × 300 μm^2^, with a total of 60 scans performed on the sample surface. The ion dose was kept smaller than 1 × 10^12^ ion/cm^2^. An electron flood gun with a target current of 2.20 pA was used for surface charging compensation during measurements. To assure the precision of spectral measurements, at least 6 data points were acquired for each sample in the positive and negative ion mode, respectively.

Analysis of the ToF-SIMS data was performed using the IONTOF Surface Lab 6.3 software (https://iontof-download.com/login.php). The mass spectra were calibrated by 15.023 CH_3_^+^, 107.075 C_4_H_11_O_3_^+^, 239.148 C_10_H_23_O_6_^+^, and 349.181 C_16_H_29_O_8_^+^ in the positive mode and 14.016 CH_2_^−^, 61.032 C_2_H_5_O_2_^−^, 105.054 C_4_H_9_O_3_^−^, and 325.185 C_14_H_29_O_8_^−^ in the negative mode, respectively. To evaluate the ability of the ToF-SIMS to distinguish masses with close *m/z* ratios. The mass deviation is defined as the difference between the observed mass and the theoretical mass divided by the theoretical mass^55,56^. It was checked by two common peaks CH_3_^+^ and CH_2_^−^ before performing mass calibration. The calculated value was smaller than 65 ppm among key peaks in all samples, suggesting that the adjacent peaks could be distinguished effectively using the high current bunched mode. The mass calibrated data were exported to Origin Pro (2019b, https://www.originlab.com/) for plotting.

**Figure 5:**
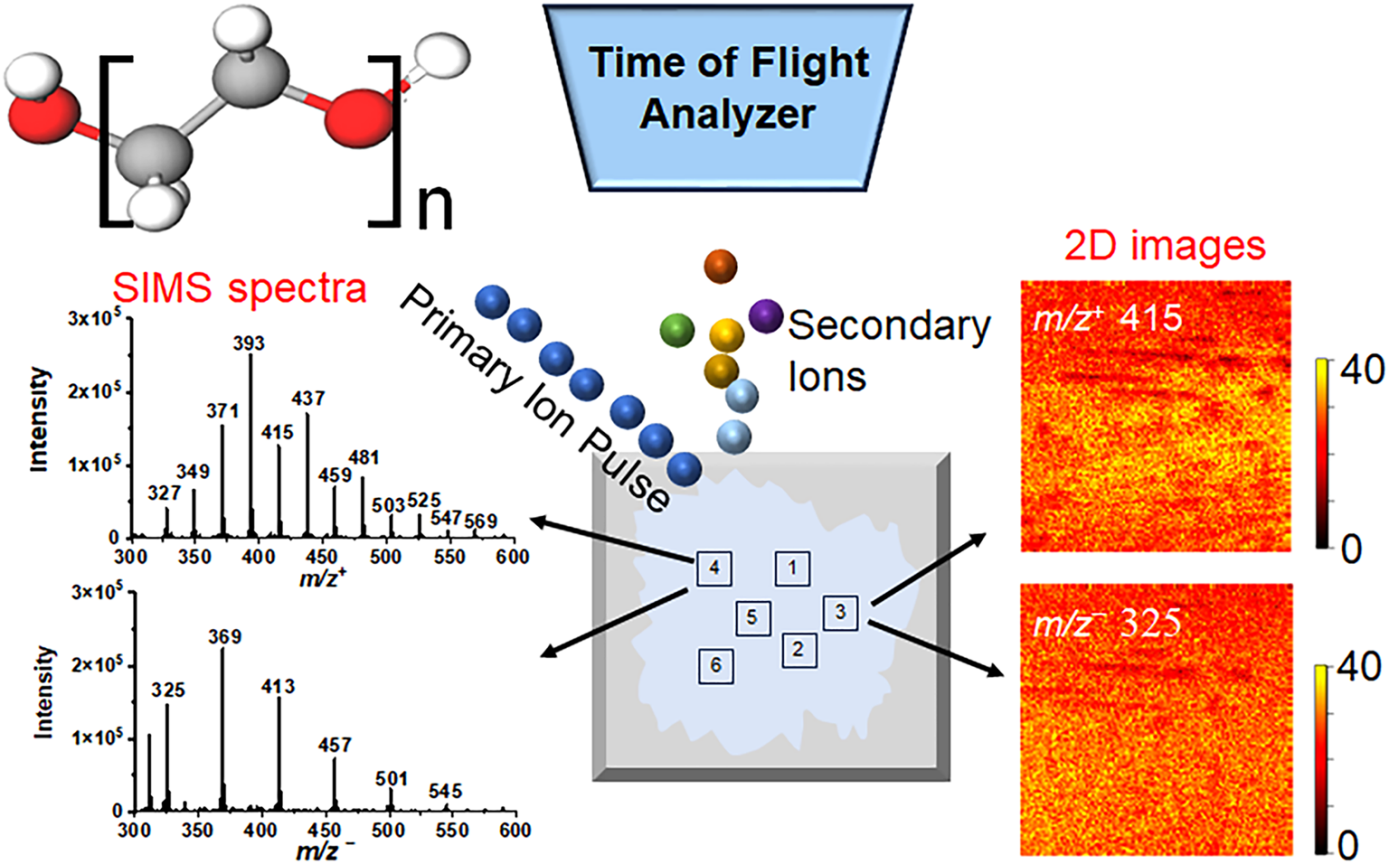
The Table of Content (TOC) graphic depicts the detection of representative PEGs using ToF-SIMS.

Additionally, spectral PCA was conducted using the Matlab software (R2021a) to investigate differences among PEG and HEG samples. The peaks of *m/z* < 50 and known PDMS interference peaks were excluded. Prior to performing PCA, the SIMS data were treated by normalization to the total ion intensities of selected peaks, square-root transformation, and mean centering^57^. ToF-SIMS 2D images^47–49^ were reconstructed using Surface Lab 6.3 software and data were exported to Igor Pro to plot the spatial distribution of various PEGs and real-world cosmetic products. Figure 5 depicts the overall workflow of PEG analysis using ToF-SIMS as the graphic highlight.

### Supplementary Information

Supplementary Information.

## Data Availability

The datasets generated for this study can be found in the article/Supplementary Material, further inquiries can be directed to the corresponding author.
