# Supplementary material for: ToF-SIMS evaluation of PEG-related mass peaks and applications in PEG detection in cosmetic products
Source: Sci Rep. 2024 Jul 1;14:14980. doi: 10.1038/s41598-024-65504-4 (PMC11217440; doi:10.1038/s41598-024-65504-4)
Supplement: Supplementary file 1 — Supplementary Information. [file 41598_2024_65504_MOESM1_ESM.docx]

Supporting Information

ToF-SIMS evaluation of PEG-related mass peaks and applications in PEG detection in cosmetic products

Yanjie Shen^1,2^, Jiyoung Son^3^, and Xiao-Ying Yu^2,*^

^1^ College of Biology and Oceanography, Weifang University, 5147 Dongfeng East Street, Weifang, Shandong 261061, China

^2^ Materials Science and Technology Division, Oak Ridge National Laboratory, Oak Ridge, TN, 37830, United States

^3^ Energy and Environment Directorate, Pacific Northwest National Laboratory, Richland, WA 99354, United States

* Correspondence: Dr. Xiao-Ying Yu, Email: yuxiaoying@ornl.gov

**Table of Contents**

[Supplementary Figures S-4](#_Toc167466939)

[**Fig. S1a.** Normalized static SIMS spectra showing reproducibility of the PEG 300 sample in the positive mode of *m/z*^+^ 0–200. S-4](#_Toc167466940)

[**Fig. S1b.** Normalized static SIMS spectra showing reproducibility of the PEG 300 sample in the positive mode of *m/z*^+^ 200–500. S-5](#_Toc167466941)

[**Fig. S1c.** Normalized static SIMS spectra showing reproducibility of the PEG 300 sample in the positive mode of *m/z*^+^ 500–800. S-6](#_Toc167466942)

[**Fig. S2a.** Static SIMS spectra showing reproducibility of the PEG 300 sample in the positive mode of *m/z*^+^ 0–200. S-7](#_Toc167466943)

[**Fig. S2b.** Static SIMS spectra showing reproducibility of the PEG 300 sample in the positive mode of *m/z*^+^ 200–500. S-8](#_Toc167466944)

[**Fig. S2c.** Static SIMS spectra showing reproducibility of the PEG 300 sample in the positive mode of *m/z*^+^ 500–800. S-9](#_Toc167466945)

[**Fig. S3a.** Normalized static SIMS spectra showing reproducibility of the PEG 300 sample in the negative mode of *m/z*^−^ 0–200. S-10](#_Toc167466946)

[**Fig. S3b.** Normalized static SIMS spectra showing reproducibility of the PEG 300 sample in the negative mode of *m/z*^−^ 200–500. S-11](#_Toc167466947)

[**Fig. S3c.** Normalized static SIMS spectra showing reproducibility of the PEG 300 sample in the negative mode of *m/z*^−^ 500–800. S-12](#_Toc167466948)

[**Fig. S4a.** Static SIMS spectra showing reproducibility of the PEG 300 sample in the negative mode of *m/z*^−^ 0–200. S-13](#_Toc167466949)

[**Fig. S4b.** Static SIMS spectra showing reproducibility of the PEG 300 sample in the negative mode of *m/z*^−^ 200–500. S-14](#_Toc167466950)

[**Fig. S4c.** Static SIMS spectra showing reproducibility of the PEG 300 sample in the negative mode of *m/z*^−^ 500–800. S-15](#_Toc167466951)

[**Fig. S5.** Normalized SIMS spectral comparison of HEG, PEGs, Clinique, and Purity in the positive mode of *m/z*^+^ 0–200. S-16](#_Toc167466952)

[**Fig. S6a.** Normalized SIMS spectral comparison of HEG, PEGs, Clinique, and Purity in the negative mode of *m/z*^−^ 0–200. S-17](#_Toc167466953)

[**Fig. S6b.** Normalized SIMS spectral comparison of HEG, PEGs, Clinique and Purity in the negative mode of *m/z*^−^ 200–500. S-18](#_Toc167466954)

[**Fig. S6c.** Normalized SIMS spectral comparison of HEG, PEGs, Clinique, and Purity in the negative mode of *m/z−* 500–800. S-19](#_Toc167466955)

[**Fig. S7.** Static SIMS spectral comparison of HEG, PEGs, Clinique, and Purity in the positive mode of *m/z*^+^ 200–500 (a) and 500–800 (b). S-21](#_Toc167466956)

[**Fig. S8.** Static SIMS spectral comparison of HEG, PEGs, Clinique, and Purity in the positive mode of *m/z*^+^ 0–200. S-22](#_Toc167466957)

[**Fig. S9a.** Static SIMS spectral comparison of HEG, PEGs, Clinique, and Purity in the negative mode of *m/z*^−^ 0–200. S-23](#_Toc167466958)

[**Fig. S9b.** Static SIMS spectral comparison of HEG, PEGs, Clinique, and Purity in the negative mode of *m/z*^−^ 200–500. S-24](#_Toc167466959)

[**Fig. S9c.** Static SIMS spectral comparison of HEG, PEGs, Clinique, and Purity in the negative mode of *m/z*^−^ 500–800. S-25](#_Toc167466960)

[**Fig. S10.** Spectral PCA results of HEG, PEGs, Clinique, and Purity in the positive mode: Scores plots of PC2 vs. PC3 and PC3 (b) loadings plot in *m/z*^+^ 50–800. Peaks are labelled in their center masses. S-26](#_Toc167466961)

[**Fig. S11.** SIMS spectral PCA results of HEG, PEGs, Clinique, and Purity in the negative mode: Scores plots of PC1 vs. PC2 (a), PC2 vs. PC4 (b) and loadings plots of PC1 (c), PC2 (d), and PC4 (e) in *m/z*^−^ 50–800. Peaks are labelled in their center masses. S-27](#_Toc167466962)

[**Fig. S12.** SIMS 2D image comparisons of key peaks (371.227, 415.252, 657.370, and 701.392) in the positive mode: (a) HEG, (b) PEG 200, (c) PEG 300, (d) PEG 400, (e) PEG 4000, (f) Purity, and (g) Clinique. S-29](#_Toc167466963)

[**Fig. S13.** Normalized SIMS 2D image comparisons of key peaks (53.001, 107.075, 129.059, and 217.106) in the positive mode: (a) HEG, (b) PEG 200, (c) PEG 300, (d) PEG 400, (e) PEG 4000, (f) Purity, and (g) Clinique. S-30](#_Toc167466964)

[**Fig. S14.** Normalized SIMS 2D image comparisons of key peaks (371.227, 415.252, 657.370, and 701.392) in the positive mode: (a) HEG, (b) PEG 200, (c) PEG 300, (d) PEG 400, (e) PEG 4000, (f) Purity, and (g) Clinique. S-31](#_Toc167466965)

[**Fig. S15.** Normalized SIMS 2D image comparisons of key peaks in the negative mode: (a) HEG, (b) PEG 200, (c) PEG 300, (d) PEG 400, (e) PEG 4000, (f) Purity, and (g) Clinique. S-32](#_Toc167466966)

[**Fig. S16.** SIMS 2D image comparisons of key peaks in the positive mode: (a) HEG, (b) PEG 200, (c) PEG 300, (d) PEG 400, (e) PEG 4000, (f) Purity, and (g) Clinique. S-33](#_Toc167466967)

[Supplementary Tables S-34](#_Toc167466968)

[**Table S1.** The HEG, PEGs, and real-word domestic products sample matrix. S-34](#_Toc167466969)

[**Table S2.** Key possible peak identification in the negative mode. S-35](#_Toc167466970)

[**Table S3.** Summary of peak area and peak height of PEG 300 representative peaks in positive ion mode. S-36](#_Toc167466971)

[**Table S4.** Ratios of peak area and peak height of PEG 300 representative peaks in positive ion mode. S-37](#_Toc167466972)

[**Table S5.** Summary of peak area and peak height of PEG 300 representative peaks in negative ion mode. S-38](#_Toc167466973)

[**Table S6.** Ratios of peak area and peak height of PEG 300 representative peaks in negative ion mode. S-39](#_Toc167466974)

[**Table S7.** Signal to noise ration of PEG 300 representative peaks in positive ion mode. S-40](#_Toc167466975)

[**Table S8.** Signal to noise ration of PEG 300 representative peaks in negative ion mode. S-41](#_Toc167466976)

[Reference S-42](#_Toc167466977)

# Supplementary Figures





**Fig. S1a.** Normalized static SIMS spectra showing reproducibility of the PEG 300 sample in the positive mode of *m/z*^+^ 0–200.

Normalized intensity (Norm. Int.) is calculated by dividing specific peak intensities to the total ion intensities of each sample, respectively. P1, P2, P3, P4, P5 and P6 represent the first, second, third, fourth, fifth and sixth positive ion mode data acquired consecutively in the PEG 300 sample.





**Fig. S1b.** Normalized static SIMS spectra showing reproducibility of the PEG 300 sample in the positive mode of *m/z*^+^ 200–500.

Normalized intensity (Norm. Int.) is calculated by dividing specific peak intensities to the total ion intensities of each sample, respectively. P1, P2, P3, P4, P5 and P6 represent the first, second, third, fourth, fifth and sixth positive ion mode data acquired consecutively in the PEG 300 sample.





**Fig. S1c.** Normalized static SIMS spectra showing reproducibility of the PEG 300 sample in the positive mode of *m/z*^+^ 500–800.

Normalized intensity (Norm. Int.) is calculated by dividing specific peak intensities to the total ion intensities of each sample, respectively. P1, P2, P3, P4, P5 and P6 represent the first, second, third, fourth, fifth and sixth positive ion mode data acquired consecutively in the PEG 300 sample.

Overall, the positive SIMS spectral comparisons of the same sample (e.g., PEG 300) in the positive ion mode show good reproducibility, thus giving assurance of data quality. The same applies to other samples we studied in this work.





**Fig. S2a.** Static SIMS spectra showing reproducibility of the PEG 300 sample in the positive mode of *m/z*^+^ 0–200.

P1, P2, P3, P4, P5 and P6 represent the first, second, third, fourth, fifth and sixth positive ion mode data acquired consecutively in the PEG 300 sample.





**Fig. S2b.** Static SIMS spectra showing reproducibility of the PEG 300 sample in the positive mode of *m/z*^+^ 200–500.

P1, P2, P3, P4, P5 and P6 represent the first, second, third, fourth, fifth and sixth positive ion mode data acquired consecutively in the PEG 300 sample.





**Fig. S2c.** Static SIMS spectra showing reproducibility of the PEG 300 sample in the positive mode of *m/z*^+^ 500–800.

P1, P2, P3, P4, P5 and P6 represent the first, second, third, fourth, fifth and sixth positive ion mode data acquired consecutively in the PEG 300 sample.

Overall, the absolute SIMS spectral comparisons of the same sample (e.g., PEG 300) in the positive ion mode are consistent with the normalized ones and show good reproducibility, thus giving assurance of data quality. The same applies to other samples we studied in this work.





**Fig. S3a.** Normalized static SIMS spectra showing reproducibility of the PEG 300 sample in the negative mode of *m/z*^−^ 0–200.

Normalized intensity (Norm. Int.) is calculated by dividing specific peak intensities to the total ion intensities of each sample, respectively. N1, N2, N3, N4, N5 and N6 represent the first, second, third, fourth, fifth and sixth negative ion mode data acquired consecutively in the PEG 300 sample.





**Fig. S3b.** Normalized static SIMS spectra showing reproducibility of the PEG 300 sample in the negative mode of *m/z*^−^ 200–500.

Normalized intensity (Norm. Int.) is calculated by dividing specific peak intensities to the total ion intensities of each sample, respectively. N1, N2, N3, N4, N5 and N6 represent the first, second, third, fourth, fifth and sixth negative ion mode data acquired consecutively in the PEG 300 sample.





**Fig. S3c.** Normalized static SIMS spectra showing reproducibility of the PEG 300 sample in the negative mode of *m/z*^−^ 500–800.

Normalized intensity (Norm. Int.) is calculated by dividing specific peak intensities to the total ion intensities of each sample, respectively. N1, N2, N3, N4, N5 and N6 represent the first, second, third, fourth, fifth and sixth negative ion mode data acquired consecutively in the PEG 300 sample.

Overall, the negative SIMS spectral comparisons of the same sample (e.g., PEG 300) in the negative ion mode show good reproducibility, thus giving assurance of data quality. The same applies to other samples we studied in this work.





**Fig. S4a.** Static SIMS spectra showing reproducibility of the PEG 300 sample in the negative mode of *m/z*^−^ 0–200.

N1, N2, N3, N4, N5 and N6 represent the first, second, third, fourth, fifth and sixth negative ion mode data acquired consecutively in the PEG 300 sample.





**Fig. S4b.** Static SIMS spectra showing reproducibility of the PEG 300 sample in the negative mode of *m/z*^−^ 200–500.

N1, N2, N3, N4, N5 and N6 represent the first, second, third, fourth, fifth and sixth negative ion mode data acquired consecutively in the PEG 300 sample.





**Fig. S4c.** Static SIMS spectra showing reproducibility of the PEG 300 sample in the negative mode of *m/z*^−^ 500–800.

N1, N2, N3, N4, N5 and N6 represent the first, second, third, fourth, fifth and sixth negative ion mode data acquired consecutively in the PEG 300 sample.

Overall, the absolute SIMS spectral comparisons of the same sample (e.g., PEG 300) in the negative ion mode are consistent with the normalized ones, and show good reproducibility, thus giving assurance of data quality. The same applies to other samples we studied in this work.





**Fig. S5.** Normalized SIMS spectral comparison of HEG, PEGs, Clinique, and Purity in the positive mode of *m/z*^+^ 0–200.

Normalized intensity (Norm. Int.) is calculated by dividing specific peak intensities to the total ion intensities of each sample, respectively. The red, blue, and green color represent different series of PEG peaks, namely red stands for HO(CH_2_CH_2_O)_n_H_2_^+^, bule stands for HO(CH_2_CH_2_O)_n_HNa^+^, and green corresponds to fragment peaks. More details of discussion can be found in main text.





**Fig. S6a.** Normalized SIMS spectral comparison of HEG, PEGs, Clinique, and Purity in the negative mode of *m/z*^−^ 0–200.

Normalized intensity (Norm. Int.) is calculated by dividing specific peak intensities to the total ion intensities of each sample, respectively. More details of discussion can be found in main text.





**Fig. S6b.** Normalized SIMS spectral comparison of HEG, PEGs, Clinique and Purity in the negative mode of *m/z*^−^ 200–500.

Normalized intensity (Norm. Int.) is calculated by dividing specific peak intensities to the total ion intensities of each sample, respectively. More details of discussion can be found in main text.





**Fig. S6c.** Normalized SIMS spectral comparison of HEG, PEGs, Clinique, and Purity in the negative mode of m/z− 500–800.

Normalized intensity (Norm. Int.) is calculated by dividing specific peak intensities to the total ion intensities of each sample, respectively. More details of discussion can be found in main text. Figures S6a-6c show similar observations to those in the positive ion mode can be found in the negative ion mode. First, a series of PEG characteristic peak are observed with reasonable peak intensities (Table S2), such as *m/z*^−^ 61.032 C_2_H_5_O_2_^−^, 105.054 C_4_H_9_O_3_^−^, 149.082 C_6_H_13_O_4_^−^, 193.108 C_8_H_17_O_5_^−^, 237.134 C_10_H_21_O_6_^−^, 281.159 C_12_H_25_O_7_^−^, 325.185 C_14_H_29_O_8_^−^, 369.216 C_16_H_33_O_9_^−^, 413.240 C_18_H_37_O_10_^−^, 457.267 C_20_H_41_O_11_^−^, 501.290 C_22_H_45_O_12_^−^, 545.290 C_24_H_49_O_13_^−^, 589.366 C_26_H_53_O_14_^−^, 633.371 C_28_H_57_O_15_^−^, and 677.397 C_30_H_61_O_16_^−^. These peaks exhibit a mass change that can be expressed by the general formula, i.e., HO(CH_2_CH_2_O)_n_^−^, where n ranges from 1 to 15. These peaks are formed when the PEG molecules HO(CH_2_CH_2_O)_n_H lose a H atom. This observation confirms that ToF-SIMS can capture the characteristic peak patterns in PEG samples. A previous study also reported ToF-SIMS could capture PEG characteristic peaks in the cross-section of ink-printed paper. However, they did not give the formula of the peaks (https://www.eag.com/app-note/imaging-cross-sections-by-tof-sims/). Second, ToF-SIMS can identify differences among PEGs. For example, the identified characteristic peak *m/z*^−^ 325.185 C_14_H_29_O_8_^−^ is observed with highest intensities in HEG and PEG 200 samples, respectively. However, other characteristic peaks, such as *m/z*^−^ 61.032 C_2_H_5_O_2_^−^, 105.054 C_4_H_9_O_3_^−^, 369.216 C_16_H_33_O_9_^−^, 413.240 C_18_H_37_O_10_^−^, 457.267 C_20_H_41_O_11_^−^, 501.290 C_22_H_45_O_12_^−^, 545.290 C_24_H_49_O_13_^−^, 589.366 C_26_H_53_O_14_^−^, and 633.371 C_28_H_57_O_15_^−^ are observed with relative high intensities in PEG 300 and 400 samples. Third, ToF-SIMS can detect PEGs in real-word cosmetic products. For instance, characteristic peaks of PEG, such as *m/z*^−^ 61.032 C_2_H_5_O_2_^−^, 193.108 C_8_H_17_O_5_^−^, 281.159 C_12_H_25_O_7_^−^, and 325.185 C_14_H_29_O_8_^−^ are observed not only in PEG samples but also in Purity and Clinique cosmetic product samples. Additionally, the peak *m/z*^−^ 193.108 C_8_H_17_O_5_^−^ is observed with relative high intensity in both Purity and Clinique samples. Interestingly, the peak *m/z*^−^ 281.159 C_12_H_25_O_7_^−^ is observed with highest intensity in Clinique sample.


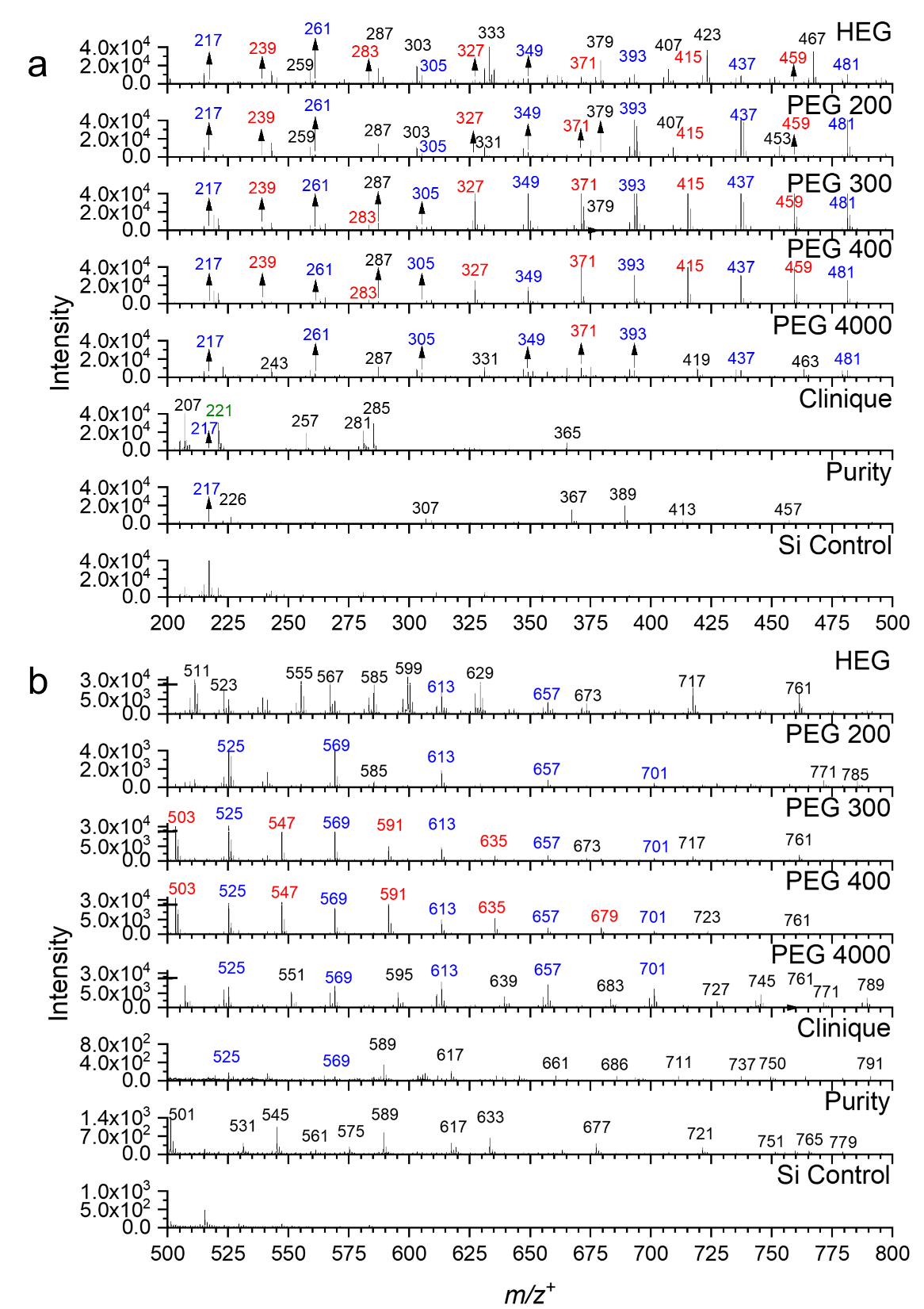


**Fig. S7.** Static SIMS spectral comparison of HEG, PEGs, Clinique, and Purity in the positive mode of *m/z*^+^ 200–500 (a) and 500–800 (b).

The red, blue, and green color represent different series of PEG peaks. Namely, red stands for HO(CH_2_CH_2_O)_n_H_2_^+^, bule stands for HO(CH_2_CH_2_O)_n_HNa^+^, and green corresponds to fragment peaks. More details of discussion can be found in main text.





**Fig. S8.** Static SIMS spectral comparison of HEG, PEGs, Clinique, and Purity in the positive mode of *m/z*^+^ 0–200.

The red, blue, and green color represent different series of PEG peaks. Namely, red stands for HO(CH_2_CH_2_O)_n_H_2_^+^, bule stands for HO(CH_2_CH_2_O)_n_HNa^+^, and green corresponds to fragment peaks. More details of discussion can be found in main text.





**Fig. S9a.** Static SIMS spectral comparison of HEG, PEGs, Clinique, and Purity in the negative mode of *m/z*^−^ 0–200.

The absolute counts of these peaks are reasonable and consistent with normalized ones.





**Fig. S9b.** Static SIMS spectral comparison of HEG, PEGs, Clinique, and Purity in the negative mode of *m/z*^−^ 200–500.

The absolute counts of these peaks are reasonable and consistent with normalized ones.





**Fig. S9c.** Static SIMS spectral comparison of HEG, PEGs, Clinique, and Purity in the negative mode of *m/z*^−^ 500–800.

The absolute counts of these peaks are reasonable and consistent with normalized ones.


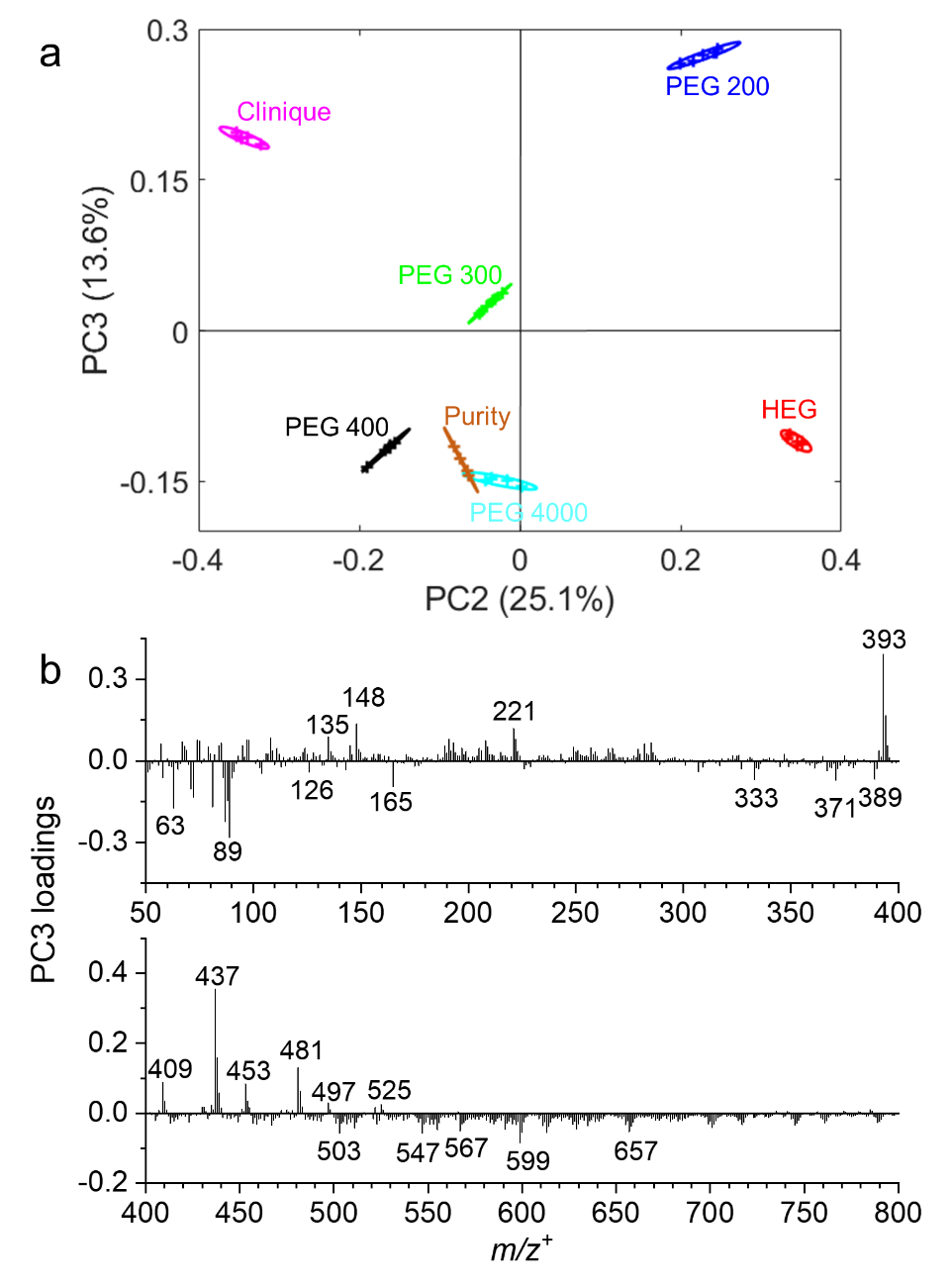


**Fig. S10.** Spectral PCA results of HEG, PEGs, Clinique, and Purity in the positive mode: Scores plots of PC2 vs. PC3 and PC3 (b) loadings plot in *m/z*^+^ 50–800. Peaks are labelled in their center masses.


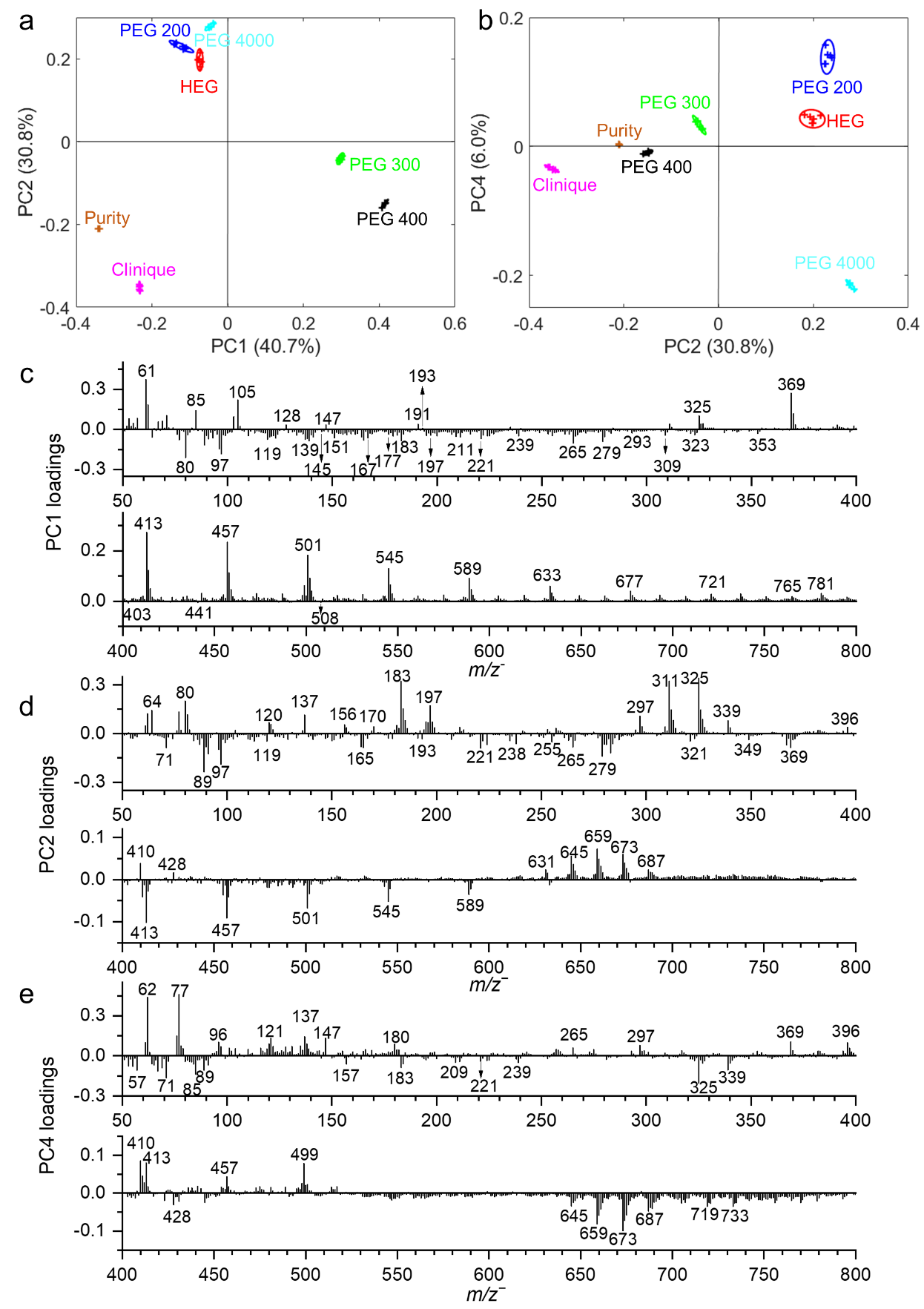


**Fig. S11.** SIMS spectral PCA results of HEG, PEGs, Clinique, and Purity in the negative mode: Scores plots of PC1 vs. PC2 (a), PC2 vs. PC4 (b) and loadings plots of PC1 (c), PC2 (d), and PC4 (e) in *m/z*^−^ 50–800. Peaks are labelled in their center masses.

Figure S11 displays scores plots of PC1 vs. PC2, PC2 vs. PC4, and corresponding principal component (PC) loadings plots in the negative mode. PC1, PC2, and PC4 can explain more than 77% of all data. Specifically, PC1 explains 40.7% of data; and it primarily separates PEG 300, and PEG 400 from HEG, PEG 200, PEG 4000, Purity, and Clinique (Fig. S11a). PC2 explains 30.8% of data; and it separates HEG, PEG 200 and PEG 4000 from PEG 300, PEG 400, Purity, and Clinique (Fig. S11a). PC4 explains 6.0% of data and separates HEG, PEG 200, PEG 300, and Purity from PEG 400, PEG 4000, and Clinique (Fig. S11b).

In PC1 positive mode loadings, PEG 300 and PEG 400 are the main contributors, indicating PEG 300 and PEG 400 share significant similarities in terms of their components. This observation is consistent with the spectral result that the most characteristic peaks are observed with relative high intensities in these two samples. The characteristic peaks, such as *m/z*^−^ 61.031 C_2_H_5_O_2_^−^, 105.054 C_4_H_9_O_3_^−^, 193.108 C_8_H_17_O_5_^−^, 325.185 C_14_H_29_O_8_^−^, 369.216 C_16_H_33_O_9_^−^, 413.240 C_18_H_37_O_10_^−^, 457.267 C_20_H_41_O_11_^−^, 501.290 C_22_H_45_O_12_^−^, 545.290 C_24_H_49_O_13_^−^, 589.366 C_26_H_53_O_14_^−^, 633.371 C_28_H_57_O_15_^−^, and 677.397 C_30_H_61_O_16_^−^ make great contributions to the variance (Fig. S11c). PC1 negative separates HEG, PEG 200, PEG 4000, Purity, and Clinique from the PEG 300 and PEG 400 (Fig. S11a), suggesting that the compositions of these sample are largely different from PEG 300 and PEG 400. Several unidentified peaks, such as *m/z*^−^ 79.970, 97.029, 151.015, 183.011, 265.079, and 279.104, display high loadings in PC1 negative mode loadings, showing that these peaks contribute more in HEG, PEG 200, PEG 4000, Purity, and Clinique.

In PC2 negative mode loadings, Purity and Clinique share common peaks with PEG 300 and PEG 400; and some of these peaks are identified as *m/z*^−^ 413.240 C_18_H_37_O_10_^−^, 457.267 C_20_H_41_O_11_^−^, 501.290 C_22_H_45_O_12_^−^, 545.290 C_24_H_49_O_13_^−^, and 589.366 C_26_H_53_O_14_^−^ (Fig. S11d). In PC2 positive mode loadings, HEG, PEG 200, and PEG 4000 share some similarities in their compositions. The identified peak *m/z*^−^ 325.185 C_14_H_29_O_8_^−^ and unidentified peaks, such as *m/z*^−^ 63.963, 79.970, 136.919, 183.011, 197.022, 311.174, 339.186, 645.316, 647.309, 659.324, and 673.338 are the main contributors.

In PC4 positive mode loadings, Purity shares some common peaks with HEG, PEG 200, and PEG 300. These peaks, including *m/z*^−^369.216 C_16_H_33_O_9_^−^, 413.240 C_18_H_37_O_10_^−^, and 457.267 C_20_H_41_O_11_^−^, are the main contributors of PC4 positive (Fig. S11e). In addition, several unidentified peaks, such as *m/z*^−^ 62.016, 77.050, 136.919, 147.071, 265.079, 297.147, 396.149, 410.151, and 499.374 make contributions. In PC4 negative mode loadings, Clinique share similarities with PEG 400 and PEG 4000, and the main contribute peaks are *m/z*^−^ 325.185 C_14_H_29_O_8_^−^, 659.324 and 673.338.


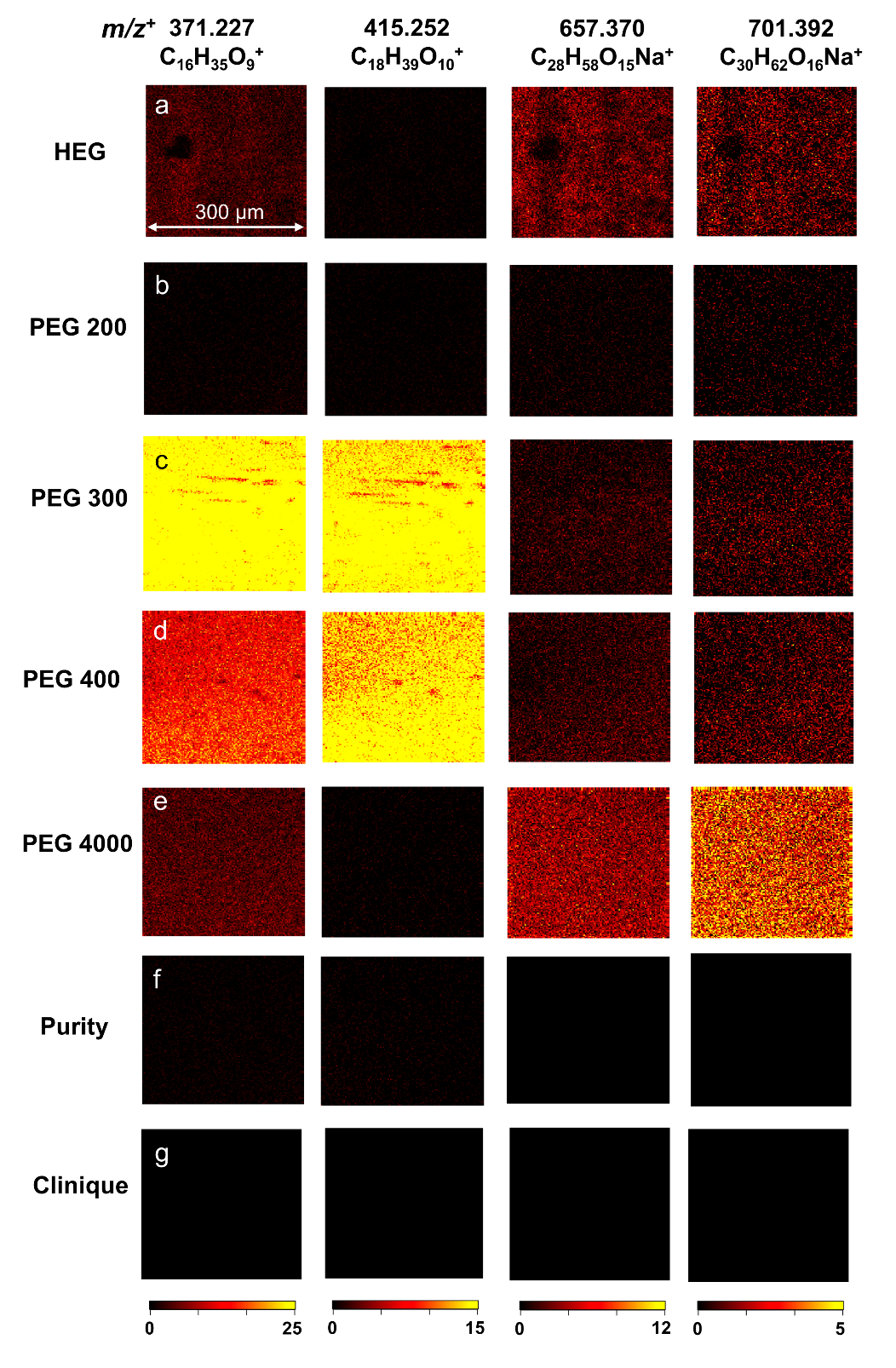


**Fig. S12.** SIMS 2D image comparisons of key peaks (371.227, 415.252, 657.370, and 701.392) in the positive mode: (a) HEG, (b) PEG 200, (c) PEG 300, (d) PEG 400, (e) PEG 4000, (f) Purity, and (g) Clinique.

The absolute counts of these peaks are reasonable and consistent with normalized ones. The counts of peaks *m/z*^+^ 657.370 and 701.392 in PEG 300 are approximately 10; however, the SNRs of them are larger than 160 (Table S7), suggesting they are real signals not noise. The same applies to other samples studied in this work. Black color in 2D images means no signals are detected.


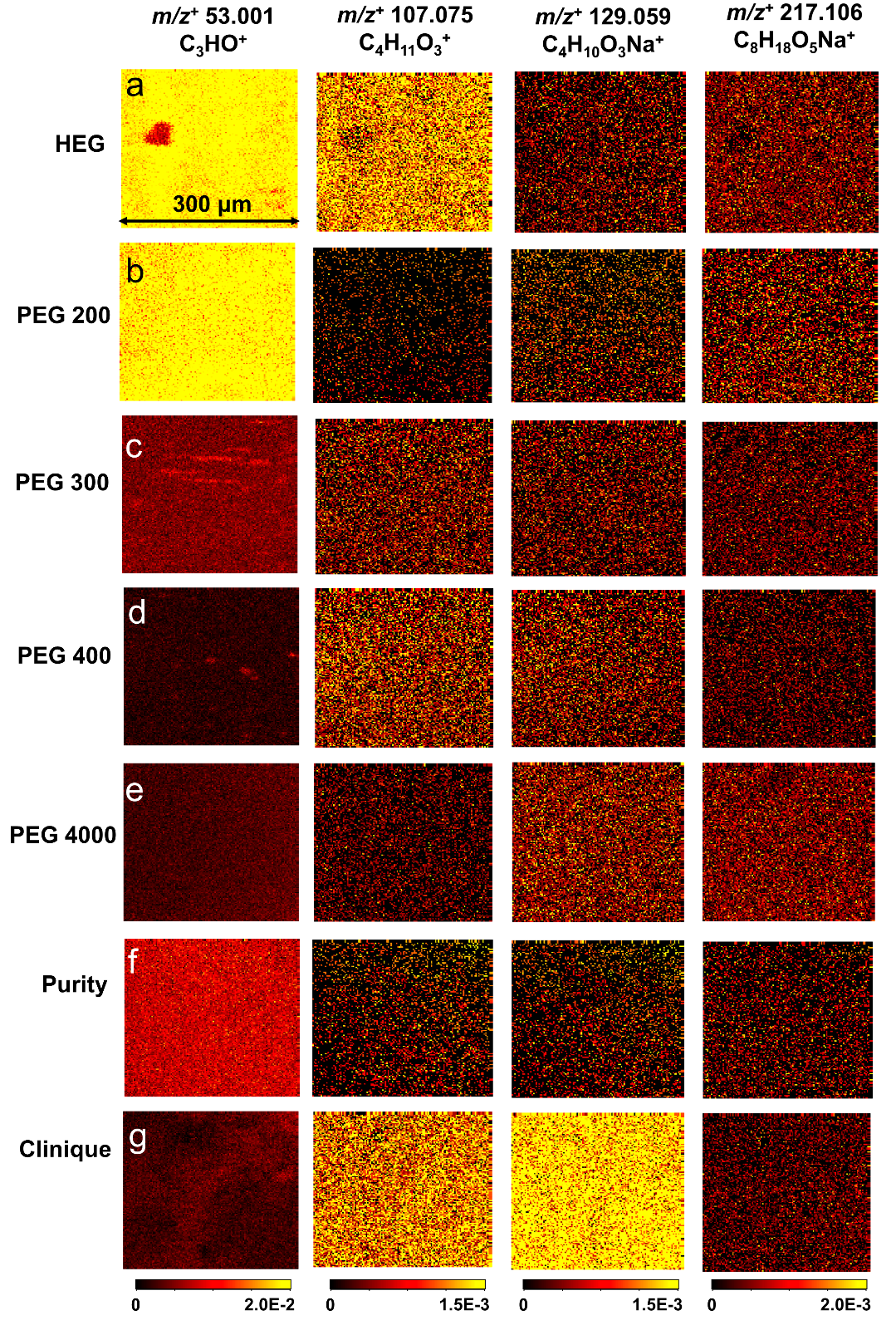


**Fig. S13.** Normalized SIMS 2D image comparisons of key peaks (53.001, 107.075, 129.059, and 217.106) in the positive mode: (a) HEG, (b) PEG 200, (c) PEG 300, (d) PEG 400, (e) PEG 4000, (f) Purity, and (g) Clinique.

The detailed discussion is illustrated in main text. Normalized intensity (Norm. Int.) is calculated by dividing specific peak intensities to the total ion intensities of each sample, respectively.


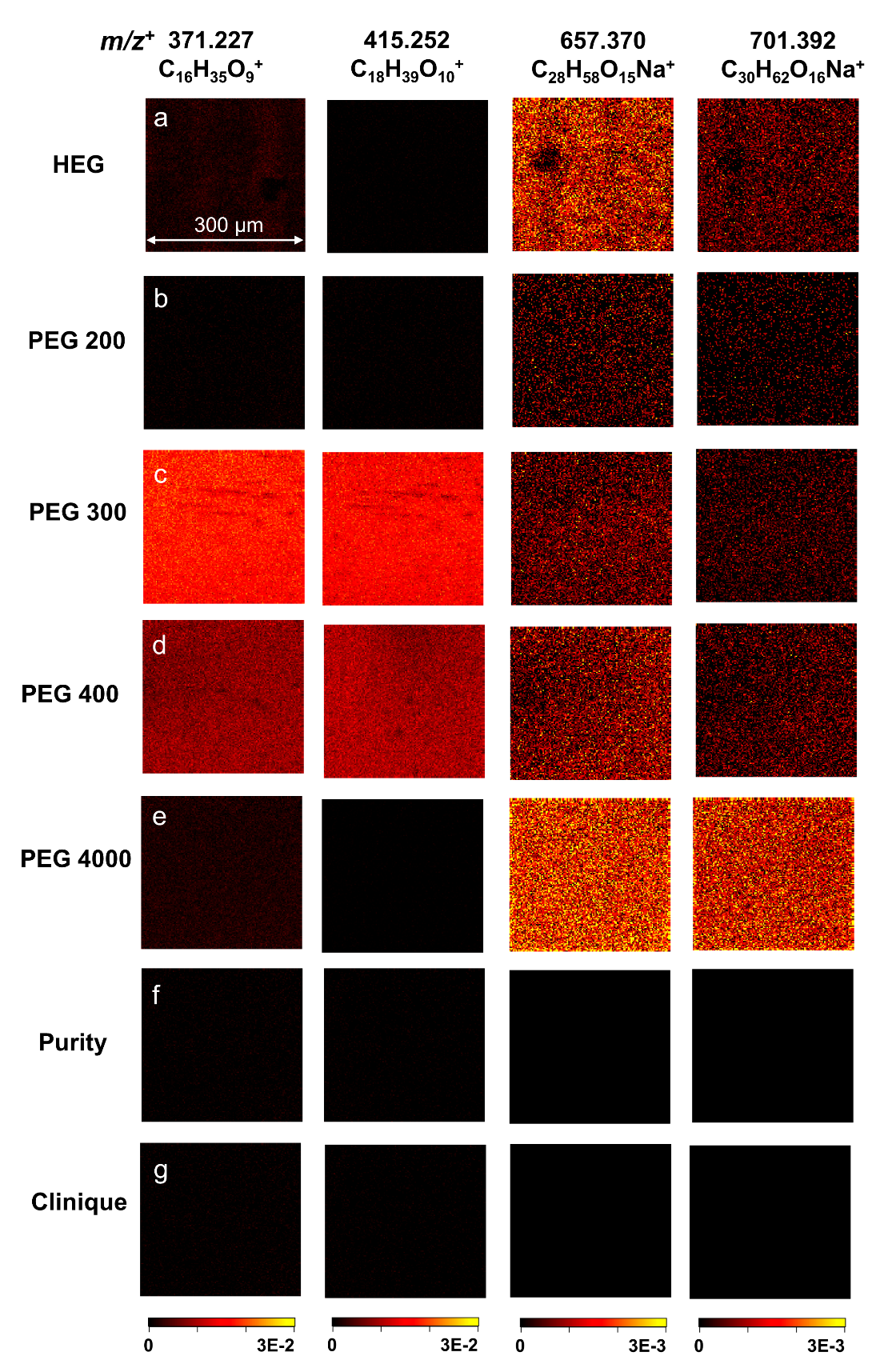


**Fig. S14.** Normalized SIMS 2D image comparisons of key peaks (371.227, 415.252, 657.370, and 701.392) in the positive mode: (a) HEG, (b) PEG 200, (c) PEG 300, (d) PEG 400, (e) PEG 4000, (f) Purity, and (g) Clinique.

The detailed discussion is illustrated in main text. Normalized intensity (Norm. Int.) is calculated by dividing specific peak intensities to the total ion intensities of each sample, respectively.


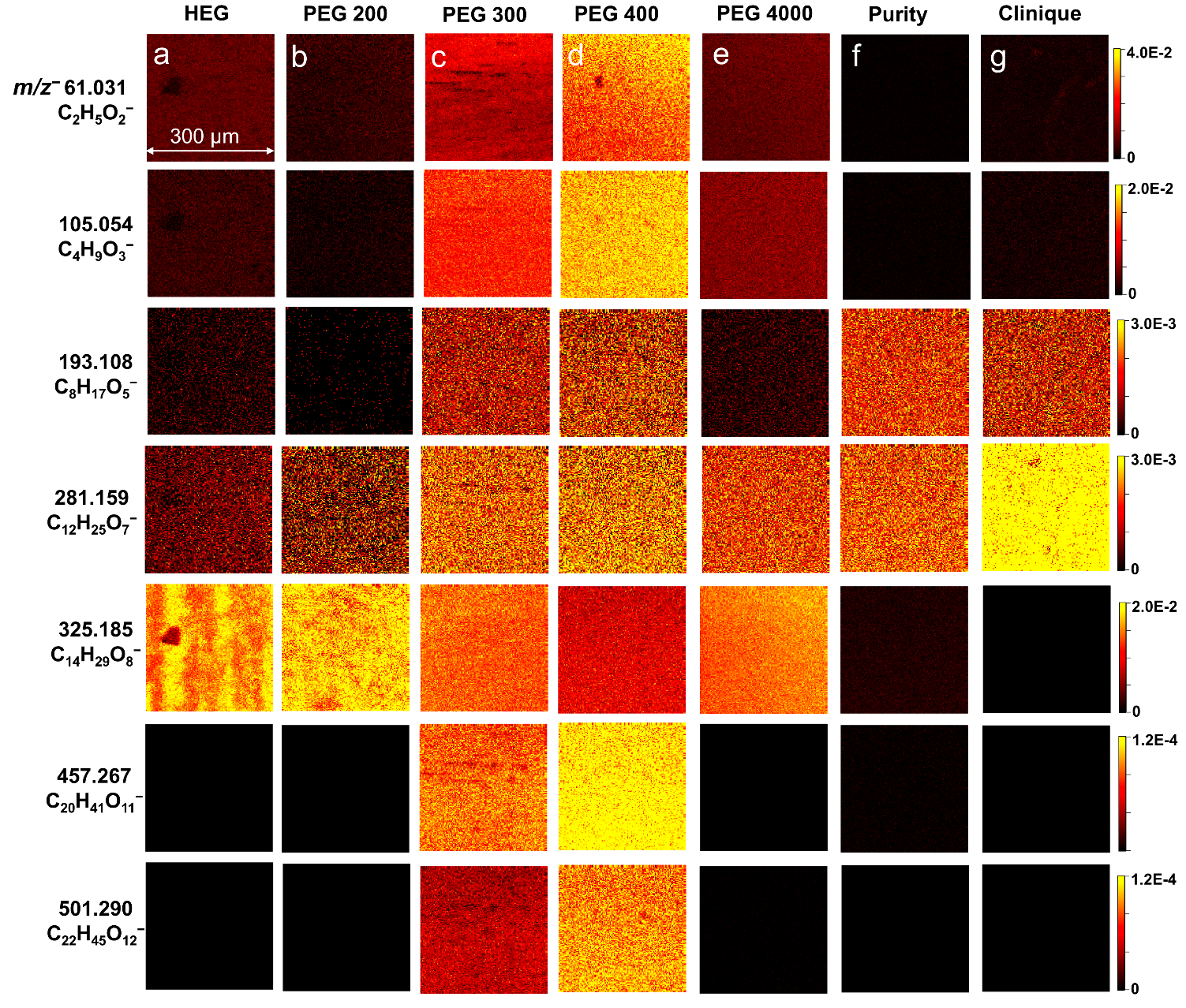


**Fig. S15.** Normalized SIMS 2D image comparisons of key peaks in the negative mode: (a) HEG, (b) PEG 200, (c) PEG 300, (d) PEG 400, (e) PEG 4000, (f) Purity, and (g) Clinique.

Normalized intensity (Norm. Int.) is calculated by dividing specific peak intensities to the total ion intensities of each sample, respectively. Figure S15 illustrates the 2D comparisons of selected key peaks observed in representative HEG, PEGs, and real-world cosmetic products in the negative ion mode. The color scale ranging from red to dark signifies higher and lower relative ion intensities, respectively. The 2D image comparison in negative ion mode shows similar results to those observed in positive ion mode. First, HEG shares some common peaks with HEGs, albeit with varying intensities, suggesting that ToF-SIMS detects the similarities and differences among samples. For example, peaks *m/z*^−^ 61.032 C_2_H_5_O_2_^−^, 105.054 C_4_H_9_O_3_^−^, 193.108 C_8_H_17_O_5_^−^, 281.159 C_12_H_25_O_7_^−^, and 325.185 C_14_H_29_O_8_^−^ are observed in HEG, PEG 200, PEG 300, PEG 400, and PEG 4000. Among these peaks, *m/z*^−^ 325.185 C_14_H_29_O_8_^−^ is more abundant in HEG, and the rest peaks are more abundant in PEGs. What’s more, peaks *m/z*^−^ 457.267 C_20_H_41_O_11_^−^ and 501.290 C_22_H_45_O_12_^−^ are detected with rich abundance only in PEG 300 and PEG 400. Second, ToF-SIMS detects PEGs in real-word cosmetic products. For instance, peaks *m/z*^−^ 193.108 C_8_H_17_O_5_^−^ and 281.159 C_12_H_25_O_7_^−^ are observed with rich abundance in Purity and Clinique.


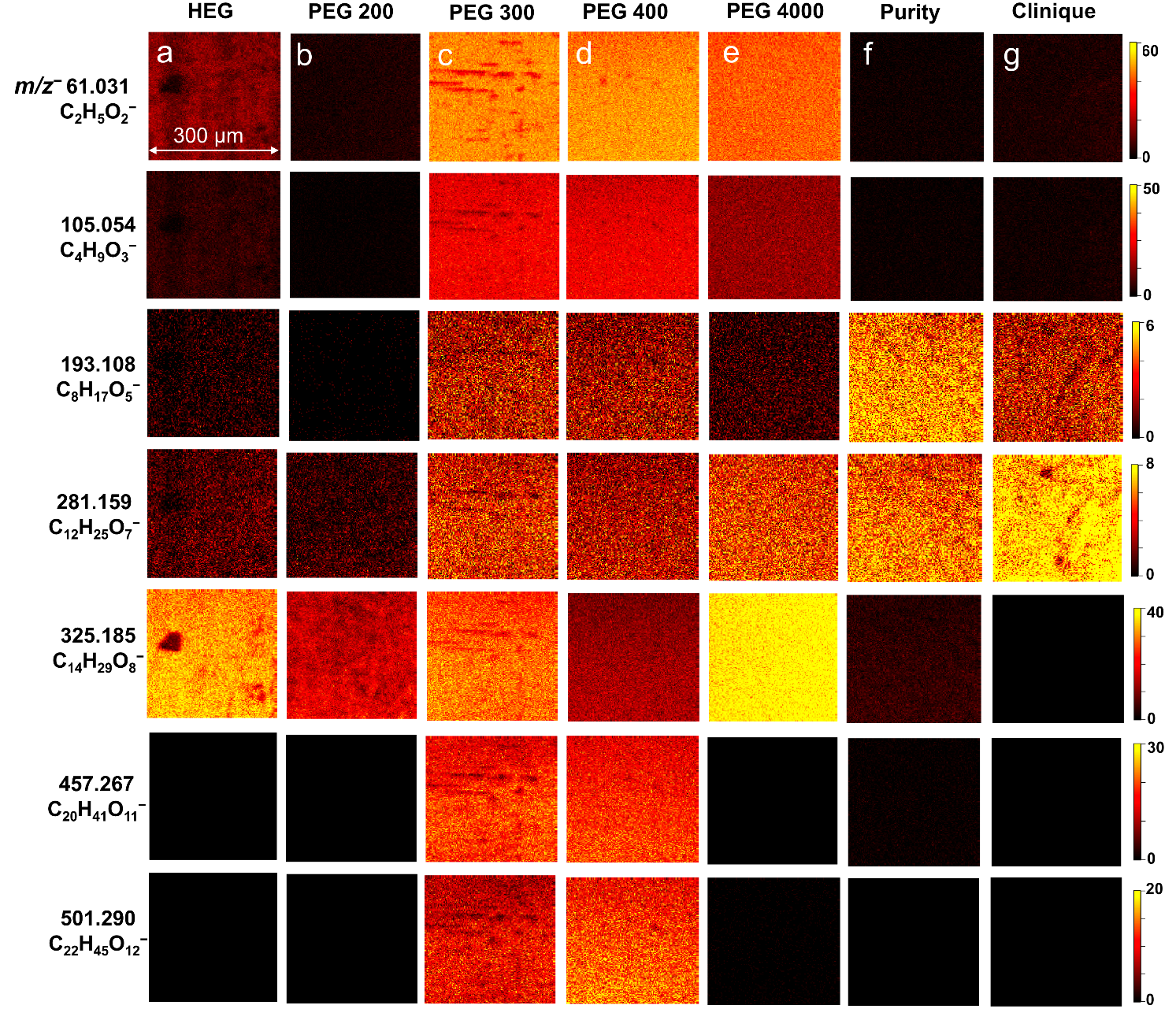


**Fig. S16.** SIMS 2D image comparisons of key peaks in the positive mode: (a) HEG, (b) PEG 200, (c) PEG 300, (d) PEG 400, (e) PEG 4000, (f) Purity, and (g) Clinique.

The absolute counts of these peaks are reasonable and consistent with normalized ones. The counts of peaks *m/z*^+^ 193.108 C_8_H_17_O_5_^−^ and 281.159 C_12_H_25_O_7_^−^ in PEG 300 are around 10, however, the SNRs of them are larger than 240 (Table S8), suggesting that they are real signals not noise. The same applies to other samples studied in this work. Black color in 2D images means no signals were detected in the surface.

# Supplementary Tables

**Table S1.** The HEG, PEGs, and real-word domestic products sample matrix.

| Sample No. | Sample name | Description |
| --- | --- | --- |
| 1 | HEG | Clear, colorless liquid |
| 2 | PEG 200 | Clear, colorless liquid |
| 3 | PEG 300 | Clear, colorless liquid |
| 4 | PEG 400 | Clear, colorless liquid |
| 5 | PEG 4000 | Milky-colored solid |
| 6 | Clinique | Yellow liquid |
| 7 | Purity | Light yellow liquid |
| 8 | Si control | Sample control |

The sample preparation was conducted in the fume hood to reduce possible air contamination. The preparation of the silicon wafer control follows existing procedures reported in our earlier publications ^1,2^.

**Table S2.** Key possible peak identification in the negative mode.

| *m/z*^−^_theo._ | *m/z*^−^_obs._ | ΔM, ppm | Formula | Description | Ref. |
| --- | --- | --- | --- | --- | --- |
| 61.028 | 61.031 | 49.158 | C_2_H_5_O_2_^−^ | HO(CH_2_CH_2_O)^−^ | This study |
| 105.055 | 105.054 | 9.519 | C_4_H_9_O_3_^−^ | HO(CH_2_CH_2_O)_2_^−^ | This study |
| 149.081 | 149.082 | 6.708 | C_6_H_13_O_4_^−^ | HO(CH_2_CH_2_O)_3_^−^ | This study |
| 193.107 | 193.108 | 5.178 | C_8_H_17_O_5_^−^ | HO(CH_2_CH_2_O)_4_^−^ | This study |
| 237.133 | 237.134 | 4.217 | C_10_H_21_O_6_^−^ | HO(CH_2_CH_2_O)_5_^−^ | This study |
| 281.159 | 281.159 | 0.000 | C_12_H_25_O_7_^−^ | HO(CH_2_CH_2_O)_6_^−^ | This study |
| 325.186 | 325.185 | 3.075 | C_14_H_29_O_8_^−^ | HO(CH_2_CH_2_O)_7_^−^ | This study |
| 369.212 | 369.216 | 2.708 | C_16_H_33_O_9_^−^ | HO(CH_2_CH_2_O)_8_^−^ | This study |
| 413.238 | 413.240 | 4.840 | C_18_H_37_O_10_^−^ | HO(CH_2_CH_2_O)_9_^−^ | This study |
| 457.264 | 457.267 | 6.561 | C_20_H_41_O_11_^−^ | HO(CH_2_CH_2_O)_10_^−^ | This study |
| 501.291 | 501.290 | 1.995 | C_22_H_45_O_12_^−^ | HO(CH_2_CH_2_O)_11_^−^ | This study |
| 545.317 | 545.290 | 49.512 | C_24_H_49_O_13_^−^ | HO(CH_2_CH_2_O)_12_^−^ | This study |
| 589.343 | 589.366 | 39.027 | C_26_H_53_O_14_^−^ | HO(CH_2_CH_2_O)_13_^−^ | This study |
| 633.369 | 633.371 | 3.158 | C_28_H_57_O_15_^−^ | HO(CH_2_CH_2_O)_14_^−^ | This study |
| 677.395 | 677.397 | 2.952 | C_30_H_61_O_16_^−^ | HO(CH_2_CH_2_O)_15_^−^ | This study |

Footnote:

*m/z*^−^_theo._: theoretical mass to charge ratio in the negative ion mode.

*m/z*^−^_obs._: observed mass to charge ratio in the negative ion mode.

ΔM:= Abs (10^6^ × (*m/z*^−^_obs._− *m/z*^−^_theo_.)/ *m/z*^−^_theo._) (expressed in ppm) ^3,4^.

**Table S3.** Summary of peak area and peak height of PEG 300 representative peaks in positive ion mode.

| *m/z*^+^_obs._^a^ | Peak Area^b^ | Area S.D.^c^ | Area RSD%^c^ | Peak height^d^ | Peak height S.D.^e^ | Peak height RSD%^e^ |
| --- | --- | --- | --- | --- | --- | --- |
| 31.019 | 4.23E+03 | 6.08E+01 | 1.44% | 4.32E+05 | 2.09E+04 | 4.84% |
| 45.035 | 2.16E+04 | 2.10E+01 | 0.10% | 2.72E+06 | 1.23E+05 | 4.52% |
| 53.001 | 8.63E+02 | 1.25E+01 | 1.45% | 5.84E+04 | 7.08E+03 | 12.13% |
| 63.045 | 3.13E+01 | 1.40E+00 | 4.49% | 2.10E+03 | 6.95E+01 | 3.32% |
| 81.074 | 3.99E+01 | 2.58E+00 | 6.47% | 1.82E+03 | 6.12E+01 | 3.36% |
| 89.064 | 1.70E+04 | 3.18E+02 | 1.87% | 5.91E+05 | 4.67E+04 | 7.91% |
| 107.075 | 6.32E+01 | 1.87E+00 | 2.95% | 2.38E+03 | 1.34E+02 | 5.63% |
| 129.059 | 7.57E+01 | 4.19E+00 | 5.54% | 1.68E+03 | 1.36E+01 | 0.81% |
| 133.094 | 5.28E+03 | 1.49E+02 | 2.82% | 1.23E+05 | 2.62E+03 | 2.13% |
| 151.098 | 8.88E+01 | 4.97E+00 | 5.60% | 2.42E+03 | 1.10E+02 | 4.55% |
| 173.081 | 6.50E+01 | 1.99E+00 | 3.06% | 1.05E+03 | 4.22E+01 | 4.03% |
| 175.103 | 1.64E+03 | 1.35E+02 | 8.23% | 3.91E+04 | 1.03E+03 | 2.63% |
| 195.123 | 1.31E+02 | 6.61E+00 | 5.04% | 3.60E+03 | 6.17E+01 | 1.72% |
| 217.106 | 9.61E+01 | 8.61E-01 | 0.90% | 1.80E+03 | 7.25E+01 | 4.03% |
| 221.150 | 8.03E+02 | 3.49E+01 | 4.34% | 1.49E+04 | 6.81E+02 | 4.58% |
| 239.148 | 2.69E+02 | 7.18E+00 | 2.67% | 4.39E+03 | 3.69E+02 | 8.40% |
| 261.128 | 1.45E+02 | 5.45E+00 | 3.76% | 2.70E+03 | 2.00E+02 | 7.40% |
| 283.176 | 4.47E+02 | 1.11E+01 | 2.49% | 6.52E+03 | 3.37E+02 | 5.17% |
| 305.151 | 2.00E+02 | 8.48E+00 | 4.24% | 3.91E+03 | 8.14E+01 | 2.08% |
| 327.201 | 2.00E+03 | 4.66E+01 | 2.33% | 3.91E+04 | 1.49E+03 | 3.80% |
| 349.181 | 3.56E+03 | 9.39E+01 | 2.63% | 6.70E+04 | 5.84E+02 | 0.87% |
| 371.227 | 9.93E+03 | 3.96E+02 | 3.99% | 1.81E+05 | 1.28E+04 | 7.10% |
| 393.210 | 1.82E+04 | 1.10E+03 | 6.04% | 3.36E+05 | 8.51E+03 | 2.53% |
| 415.252 | 9.19E+03 | 3.88E+02 | 4.22% | 1.48E+05 | 9.11E+03 | 6.14% |
| 437.238 | 1.53E+04 | 3.17E+02 | 2.08% | 2.41E+05 | 8.90E+03 | 3.70% |
| 459.280 | 6.38E+03 | 1.75E+02 | 2.74% | 8.83E+04 | 4.72E+03 | 5.34% |
| 481.263 | 8.74E+03 | 2.04E+02 | 2.34% | 1.19E+05 | 1.51E+03 | 1.27% |
| 503.307 | 3.26E+03 | 1.31E+02 | 4.02% | 3.95E+04 | 1.83E+03 | 4.64% |
| 525.293 | 4.15E+03 | 8.28E+01 | 2.00% | 4.57E+04 | 6.46E+02 | 1.41% |
| 547.300 | 1.28E+03 | 6.01E+01 | 4.70% | 1.31E+04 | 6.50E+02 | 4.97% |
| 569.281 | 1.71E+03 | 5.10E+01 | 2.99% | 1.67E+04 | 4.07E+02 | 2.44% |
| 591.354 | 4.90E+02 | 2.43E+01 | 4.96% | 6.42E+03 | 2.50E+02 | 3.89% |
| 613.352 | 7.95E+02 | 2.12E+01 | 2.67% | 6.41E+03 | 1.82E+02 | 2.84% |
| 635.399 | 2.69E+02 | 7.95E+00 | 2.95% | 2.05E+03 | 6.76E+01 | 3.30% |
| 657.367 | 3.18E+02 | 6.37E+00 | 2.00% | 2.43E+03 | 5.51E+01 | 2.27% |
| 679.417 | 1.22E+02 | 4.94E+00 | 4.04% | 8.56E+02 | 5.91E+01 | 6.90% |
| 701.392 | 1.72E+02 | 5.51E+00 | 3.21% | 1.16E+03 | 4.71E+01 | 4.05% |

Footnote:

^a^: *m/z*^+^_obs._: observed mass to charge ratio in the positive ion mode.

^b^: average peak area of three replicate samples.

^c^: standard deviation (S.D.) and relative standard deviation (RSD%) of peak area. Relative standard deviation (RSD%)=S.D./Average×100%.

^d^: average peak height of four replicate samples.

^e^: standard deviation (S.D.) and relative standard deviation (RSD%) of peak height.

**Table S4.** Ratios of peak area and peak height of PEG 300 representative peaks in positive ion mode.

| *m/z^+^*_obs._ | Peak Area ratio^a^ | Area ratio S.D.^b^ | Area ratio RSD%^b^ | Peak height ratio^d^ | Height ratio S.D.^e^ | Peak height ratio RSD%^f^ |
| --- | --- | --- | --- | --- | --- | --- |
| 31.019 | 0.0366 | 0.0007 | 1.91% | 0.1657 | 0.0037 | 2.23% |
| 45.033 | 0.1556 | 0.0019 | 1.22% | 0.5065 | 0.0146 | 2.88% |
| 53.001 | 0.0075 | 0.0002 | 2.67% | 0.0224 | 0.0021 | 9.38% |
| 63.045 | 0.0003 | 0.0000 | 0.00% | 0.0008 | 0.0000 | 0.00% |
| 81.074 | 0.0003 | 0.0000 | 0.00% | 0.0007 | 0.0000 | 0.00% |
| 89.064 | 0.1468 | 0.0041 | 2.79% | 0.2264 | 0.0118 | 5.21% |
| 107.075 | 0.0005 | 0.0000 | 0.00% | 0.0009 | 0.0001 | 11.11% |
| 129.059 | 0.0007 | 0.0000 | 0.00% | 0.0006 | 0.0000 | 0.00% |
| 133.094 | 0.0457 | 0.0014 | 3.06% | 0.0472 | 0.0015 | 3.18% |
| 151.098 | 0.0008 | 0.0000 | 0.00% | 0.0009 | 0.0000 | 0.00% |
| 173.081 | 0.0006 | 0.0000 | 0.00% | 0.0004 | 0.0000 | 0.00% |
| 175.096 | 0.0118 | 0.0009 | 7.63% | 0.0073 | 0.0003 | 4.11% |
| 195.123 | 0.0011 | 0.0001 | 9.09% | 0.0014 | 0.0000 | 0.00% |
| 217.106 | 0.0008 | 0.0000 | 0.00% | 0.0007 | 0.0000 | 0.00% |
| 221.150 | 0.0069 | 0.0003 | 4.35% | 0.0057 | 0.0003 | 5.26% |
| 239.148 | 0.0023 | 0.0000 | 0.00% | 0.0017 | 0.0001 | 5.88% |
| 261.128 | 0.0013 | 0.0000 | 0.00% | 0.0010 | 0.0001 | 10.00% |
| 283.176 | 0.0039 | 0.0001 | 2.56% | 0.0025 | 0.0001 | 4.00% |
| 305.151 | 0.0017 | 0.0001 | 5.88% | 0.0015 | 0.0000 | 0.00% |
| 327.201 | 0.0173 | 0.0004 | 2.31% | 0.0150 | 0.0009 | 6.00% |
| 349.181 | 0.0308 | 0.0004 | 1.30% | 0.0257 | 0.0006 | 2.33% |
| 371.227 | 0.0859 | 0.0025 | 2.91% | 0.0692 | 0.0040 | 5.78% |
| 393.210 | 0.1572 | 0.0075 | 4.77% | 0.1289 | 0.0061 | 4.73% |
| 415.252 | 0.0795 | 0.0030 | 3.77% | 0.0570 | 0.0046 | 8.07% |
| 437.238 | 0.1321 | 0.0026 | 1.97% | 0.0924 | 0.0053 | 5.74% |
| 459.280 | 0.0552 | 0.0022 | 3.99% | 0.0339 | 0.0010 | 2.95% |
| 481.263 | 0.0756 | 0.0017 | 2.25% | 0.0458 | 0.0015 | 3.28% |
| 503.307 | 0.0282 | 0.0014 | 4.96% | 0.0152 | 0.0004 | 2.63% |
| 525.293 | 0.0359 | 0.0007 | 1.95% | 0.0175 | 0.0006 | 3.43% |
| 547.300 | 0.0110 | 0.0006 | 5.45% | 0.0050 | 0.0001 | 2.00% |
| 569.281 | 0.0148 | 0.0004 | 2.70% | 0.0064 | 0.0003 | 4.69% |
| 591.354 | 0.0042 | 0.0002 | 4.76% | 0.0025 | 0.0001 | 4.00% |
| 613.352 | 0.0069 | 0.0001 | 1.45% | 0.0025 | 0.0001 | 4.00% |
| 635.399 | 0.0023 | 0.0001 | 4.35% | 0.0008 | 0.0000 | 0.00% |
| 657.367 | 0.0028 | 0.0000 | 0.00% | 0.0009 | 0.0000 | 0.00% |
| 679.417 | 0.0011 | 0.0000 | 0.00% | 0.0003 | 0.0000 | 0.00% |
| 701.392 | 0.0015 | 0.0000 | 0.00% | 0.0004 | 0.0000 | 0.00% |

Footnote:

^a^: average peak area ratio between the counts of specific peaks and total counts of all key products/fragments

^b^: standard deviation (S.D.) of peak area ratio.

^c^: Relative standard deviation (RSD%)=S.D./Average×100%, RSD of the peak area ratio.

^d^: average peak height ratio of three replicate samples.

^e^: standard deviation (S.D.) of the peak height ratio.

^f^: RSD of peak height ratio.

**Table S5.** Summary of peak area and peak height of PEG 300 representative peaks in negative ion mode.

| *m/z*^−^_obs._^a^ | Peak Area^b^ | Area S.D.^c^ | Area RSD%^c^ | Peak Height^d^ | Peak height S.D.^e^ | Peak height RSD%^e^ |
| --- | --- | --- | --- | --- | --- | --- |
| 61.031 | 1.03E+04 | 1.45E+02 | 1.41% | 4.03E+05 | 9.25E+03 | 2.30% |
| 105.054 | 4.25E+03 | 4.51E+01 | 1.06% | 1.24E+05 | 4.15E+03 | 3.35% |
| 149.082 | 4.34E+02 | 8.71E+00 | 2.01% | 9.06E+03 | 3.00E+02 | 3.31% |
| 193.108 | 4.83E+02 | 1.40E+01 | 2.90% | 8.77E+03 | 4.16E+02 | 4.74% |
| 237.134 | 7.22E+02 | 5.95E+00 | 0.82% | 1.22E+04 | 4.65E+02 | 3.81% |
| 281.159 | 1.02E+03 | 3.10E+01 | 3.04% | 1.42E+04 | 6.89E+02 | 4.85% |
| 325.185 | 8.87E+03 | 5.49E+02 | 6.19% | 1.38E+05 | 7.81E+03 | 5.66% |
| 369.216 | 1.19E+04 | 6.15E+02 | 5.17% | 2.15E+05 | 1.17E+04 | 5.44% |
| 413.240 | 9.55E+03 | 5.24E+02 | 5.49% | 1.38E+05 | 1.11E+04 | 8.04% |
| 457.267 | 4.93E+03 | 1.16E+01 | 0.24% | 6.97E+04 | 4.50E+03 | 6.46% |
| 501.290 | 2.36E+03 | 1.42E+02 | 6.02% | 2.60E+04 | 1.28E+02 | 0.49% |
| 545.290 | 1.00E+03 | 5.30E+01 | 5.30% | 9.34E+03 | 8.11E+02 | 8.68% |
| 589.366 | 4.04E+02 | 1.71E+01 | 4.23% | 4.09E+03 | 2.54E+02 | 6.21% |
| 633.371 | 1.72E+02 | 3.52E+00 | 2.05% | 1.73E+03 | 9.98E+01 | 5.77% |
| 677.397 | 1.02E+02 | 8.39E+00 | 8.23% | 8.92E+02 | 6.74E+01 | 7.56% |

Footnote:

^a^: *m/z*^−^_obs.._: observed mass to charge ratio in the negative ion mode.

^b^: average peak area of three replicate samples.

^c^: standard deviation (S.D.) and relative standard deviation (RSD%) of peak area. Relative standard deviation (RSD%)=S.D./Average×100%.

^d^: average peak height of four replicate samples.

^e^: standard deviation (S.D.) and relative standard deviation (RSD%) of peak height.

**Table S6.** Ratios of peak area and peak height of PEG 300 representative peaks in negative ion mode.

| *m/z*^−^_obs._ | Peak Area ratio^a^ | Area ratio S.D.^b^ | Area ratio RSD%^c^ | Peak height ratio^d^ | Height ratio S.D.^e^ | Peak height ratio RSD%^f^ |
| --- | --- | --- | --- | --- | --- | --- |
| 61.031 | 0.1817 | 0.0031 | 1.71% | 0.3435 | 0.0060 | 1.75% |
| 105.054 | 0.0752 | 0.0014 | 1.86% | 0.1057 | 0.0033 | 3.12% |
| 149.082 | 0.0077 | 0.0001 | 1.30% | 0.0077 | 0.0001 | 1.30% |
| 193.108 | 0.0086 | 0.0004 | 4.65% | 0.0075 | 0.0002 | 2.67% |
| 237.134 | 0.0128 | 0.0004 | 3.13% | 0.0104 | 0.0002 | 1.92% |
| 281.159 | 0.0180 | 0.0002 | 1.11% | 0.0120 | 0.0003 | 2.50% |
| 325.185 | 0.1571 | 0.0092 | 5.86% | 0.1174 | 0.0059 | 5.03% |
| 369.216 | 0.2111 | 0.0057 | 2.70% | 0.1834 | 0.0056 | 3.05% |
| 413.240 | 0.1690 | 0.0056 | 3.31% | 0.1172 | 0.0097 | 8.28% |
| 457.267 | 0.0874 | 0.0024 | 2.75% | 0.0593 | 0.0024 | 4.05% |
| 501.290 | 0.0418 | 0.0014 | 3.35% | 0.0221 | 0.0005 | 2.26% |
| 545.290 | 0.0178 | 0.0006 | 3.37% | 0.0079 | 0.0005 | 6.33% |
| 589.366 | 0.0071 | 0.0002 | 2.82% | 0.0035 | 0.0002 | 5.71% |
| 633.371 | 0.0031 | 0.0001 | 3.23% | 0.0015 | 0.0000 | 0.00% |
| 677.397 | 0.0018 | 0.0001 | 5.56% | 0.0008 | 0.0000 | 0.00% |

Footnote:

^a^: average peak area ratio between the counts of specific peaks and total counts of all key products/fragments.

^b^: standard deviation (S.D.) of peak area ratio.

^c^: Relative standard deviation (RSD%)=S.D./Average×100%, RSD of the peak area ratio.

^d^: average peak height ratio of three replicate samples.

^e^: standard deviation (S.D.) of the peak height ratio.

^f^: RSD of peak height ratio.

**Table S7.** Signal to noise ration of PEG 300 representative peaks in positive ion mode.

| *m/z*^+^_obs._ | Formula | SNR^a^ |
| --- | --- | --- |
| 31.019 | CH_3_O^+^ | 61181.57 |
| 45.035 | C_2_H_5_O^+^ | 24762.88 |
| 53.001 | C_3_HO^+^ | 2360.79 |
| 63.045 | C_2_H_7_O_2_^+^ | 47.79 |
| 81.074 | C_6_H_9_^+^ | 45.31 |
| 89.064 | C_4_H_9_O_2_^+^ | 18901.72 |
| 107.075 | C_4_H_11_O_3_^+^ | 65.58 |
| 129.059 | C_6_H_9_O_3_^+^ | 36.56 |
| 133.094 | C_6_H_13_O_3_^+^ | 2637.15 |
| 151.098 | C_6_H_15_O_4_^+^ | 101.62 |
| 173.081 | C_8_H_13_O_4_^+^ | 39.91 |
| 175.103 | C_8_H_15_O_4_^+^ | 1789.73 |
| 195.123 | C_8_H_19_O_5_^+^ | 149.86 |
| 217.106 | C_10_H_17_O_5_^+^ | 43.68 |
| 221.150 | C_14_H_21_O_2_^+^ | 385.41 |
| 239.148 | C_10_H_23_O_6_^+^ | 97.20 |
| 261.128 | C_12_H_21_O_6_^+^ | 46.96 |
| 283.176 | C_12_H_27_O_7_^+^ | 121.60 |
| 305.151 | C_14_H_25_O_7_^+^ | 60.84 |
| 327.201 | C_14_H_31_O_8_^+^ | 815.46 |
| 349.181 | C_16_H_29_O_8_^+^ | 1442.41 |
| 371.227 | C_16_H_35_O_9_^+^ | 3266.17 |
| 393.210 | C_18_H_33_O_9_^+^ | 5961.19 |
| 415.252 | C_18_H_39_O_10_^+^ | 4046.90 |
| 437.238 | C_20_H_37_O_10_^+^ | 5004.03 |
| 459.280 | C_20_H_43_O_11_^+^ | 2501.32 |
| 481.263 | C_22_H_41_O_11_^+^ | 3337.48 |
| 503.307 | C_22_H_47_O_12_^+^ | 1912.00 |
| 525.293 | C_24_H_45_O_12_^+^ | 1614.50 |
| 547.300 | C_24_H_51_O_13_^+^ | 1057.20 |
| 569.281 | C_26_H_49_O_13_^+^ | 743.69 |
| 591.354 | C_26_H_55_O_14_^+^ | 551.89 |
| 613.352 | C_28_H_53_O_14_^+^ | 455.30 |
| 635.399 | C_28_H_59_O_15_^+^ | 319.60 |
| 657.367 | C_30_H_57_O_15_^+^ | 848.00 |
| 679.417 | C_30_H_63_O_16_^+^ | 165.25 |
| 701.392 | C_32_H_61_O_16_^+^ | 168.40 |

Footnote:

^a^ SNR: signal to noise ratio.

**Table S8.** Signal to noise ration of PEG 300 representative peaks in negative ion mode.

| *m/z*^−^_obs._ | Formula | SNR^a^ |
| --- | --- | --- |
| 61.031 | C_2_H_5_O_2_^−^ | 17996.30 |
| 105.054 | C_4_H_9_O_3_^−^ | 9166.14 |
| 149.082 | C_6_H_13_O_4_^−^ | 666.71 |
| 193.108 | C_8_H_17_O_5_^−^ | 246.32 |
| 237.134 | C_10_H_21_O_6_^−^ | 376.00 |
| 281.159 | C_12_H_25_O_7_^−^ | 401.32 |
| 325.185 | C_14_H_29_O_8_^−^ | 2207.52 |
| 369.216 | C_16_H_33_O_9_^−^ | 2987.04 |
| 413.240 | C_18_H_37_O_10_^−^ | 2306.15 |
| 457.267 | C_20_H_41_O_11_^−^ | 1454.48 |
| 501.290 | C_22_H_45_O_12_^−^ | 772.69 |
| 545.290 | C_24_H_49_O_13_^−^ | 407.00 |
| 589.366 | C_26_H_53_O_14_^−^ | 218.72 |
| 633.371 | C_28_H_57_O_15_^−^ | 87.10 |
| 677.397 | C_30_H_61_O_16_^−^ | 45.95 |

Footnote:

^a^ SNR: signal to noise ratio.

# Reference

1 Fu, Y. *et al.* Does interfacial photochemistry play a role in the photolysis of pyruvic acid in water? *Atmos. Environ.* **191**, 36-45, doi:10.1016/j.atmosenv.2018.07.061 (2018).

2 Sui, X. *et al.* ToF‐SIMS characterization of glyoxal surface oxidation products by hydrogen peroxide: A comparison between dry and liquid samples. *Surf. Interface Anal.* **50**, 927-938, doi:10.1002/sia.6334 (2018).

3 Gilmore, I. S. & Seah, M. P. Static SIMS: towards unfragmented mass spectra — the G-SIMS procedure. *Appl. Surf. Sci.* **161**, 465-480, doi:10.1016/s0169-4332(00)00317-2 (2000).

4 Yu, X.-Y. *et al.* Molecular detection of per- and polyfluoroalkyl substances in water using time-of-flight secondary ion mass spectrometry. *Front. Chem.* **11**, 1253685, doi:10.3389/fchem.2023.1253685 (2023).
